# Bioassay-Guided Fractionation Leads to the Detection of Cholic Acid Generated by the Rare *Thalassomonas* sp.

**DOI:** 10.3390/md21010002

**Published:** 2022-12-20

**Authors:** Fazlin Pheiffer, Yannik K.-H. Schneider, Espen Holst Hansen, Jeanette Hammer Andersen, Johan Isaksson, Tobias Busche, Christian Rückert, Jörn Kalinowski, Leonardo van Zyl, Marla Trindade

**Affiliations:** 1 Institute for Microbial Biotechnology and Metagenomics (IMBM), Department of Biotechnology, University of the Western Cape, Robert Sobukwe Road, Bellville, Cape Town 7535, South Africa; 2 Marbio, UiT—The Arctic University of Norway, N-9037 Tromsø, Norway; 3 Department of Chemistry, UiT —The Arctic University of Norway, Breivika, N-9037 Tromsø, Norway; 4 Department of Pharmacy, UiT—The Arctic University of Norway, Breivika, N-9037 Tromsø, Norway; 5 Institute for Innovation Transfer GmbH (IIT), Universitätsstraße 25, 33615 Bielefeld, Germany

**Keywords:** *Thalassomonas*, cholic acid, bioactivity, genome mining, bile acid, bacterial natural products, homoserine lactone acylase

## Abstract

Bacterial symbionts of marine invertebrates are rich sources of novel, pharmaceutically relevant natural products that could become leads in combatting multidrug-resistant pathogens and treating disease. In this study, the bioactive potential of the marine invertebrate symbiont *Thalassomonas actiniarum* was investigated. Bioactivity screening of the strain revealed Gram-positive specific antibacterial activity as well as cytotoxic activity against a human melanoma cell line (A2058). The dereplication of the active fraction using HPLC-MS led to the isolation and structural elucidation of cholic acid and 3-oxo cholic acid. *T. actiniarum* is one of three type species belonging to the genus *Thalassomonas*. The ability to generate cholic acid was assessed for all three species using thin-layer chromatography and was confirmed by LC-MS. The re-sequencing of all three *Thalassomonas* type species using long-read Oxford Nanopore Technology (ONT) and Illumina data produced complete genomes, enabling the bioinformatic assessment of the ability of the strains to produce cholic acid. Although a complete biosynthetic pathway for cholic acid synthesis in this genus could not be determined based on sequence-based homology searches, the identification of putative penicillin or homoserine lactone acylases in all three species suggests a mechanism for the hydrolysis of conjugated bile acids present in the growth medium, resulting in the generation of cholic acid and 3-oxo cholic acid. With little known currently about the bioactivities of this genus, this study serves as the foundation for future investigations into their bioactive potential as well as the potential ecological role of bile acid transformation, sterol modification and quorum quenching by *Thalassomonas* sp. in the marine environment.

## 1. Introduction

With the advent of multidrug-resistant (MDR) pathogens and the emergence of new infectious diseases, the search for new drugs has become of utmost importance. Natural products (NPs) have been used since ancient times to treat diseases and are the most successful sources of new drug candidates [1,2], with the majority of these being NPs or derivatives of NPs [3,4]. Marine bioprospecting has contributed significantly to the discovery of novel bioactive NPs with unique structures and biological activities, offering alternatives to compounds from terrestrial origin, against which resistance has developed [5]. Marine invertebrate symbionts are particularly promising sources of marine NPs, as the competition between microorganisms associated with invertebrates for space and nutrients is the driving force behind the evolution and production of antagonistic compounds, which often constitute pharmaceutically relevant natural products [6].

Top-down approaches towards the discovery of NPs have traditionally been the primary means of NP discovery, and, to date, this is still a successful strategy. However, genome-guided strategies are being incorporated as alternative or parallel approaches to NP discovery, as they allow for the investigation of the biosynthetic potential and metabolic capacity of microorganisms rather than just what can be detected in assays [7,8,9,10]. In addition, by combining integrative omics and bioinformatic analysis, insight into the activation of BGCs that are silent under standard laboratory culturing conditions could be provided. Genome-mining tools can be used to identify novel gene clusters, and through the implementation of genetic engineering techniques, these can be activated to produce the natural products they encode [11,12,13,14].

Here, we describe the completion of the genomes of the three poorly characterized *Thalassomonas* species and perform genome mining to assess their bioactive potential. We further demonstrate that all three species are capable of the generation of cholic acid when cultured in marine broth, a bile acid known to inhibit the growth of gram-positive bacterial strains [15,16]. Furthermore, we identify putative homoserine lactone acylases, which are promising biocatalysts with a wide range of applications in the health, agricultural and aquaculture industries.

## 2. Results

### 2.1. Extract Screening for Antimicrobial Activity and Cytotoxicity

To investigate the bioactivity of metabolites produced by *T. actiniarum*, extracts were prepared, as described in the Materials and Methods section, which resulted in approximately 1.5 g of dried crude extract. Following flash fractionation, six fractions were generated. Fractions 1–5 were screened for antibacterial activity (250 µg/mL) and cytotoxicity (100 µg/mL). Due to the paucity of the material obtained in Fraction 6, it was not tested. Fraction 5 displayed activity against the Gram-positive test strains *Enterococcus faecalis*, *Staphylococcus aureus* and *Streptococcus agalactiae* (OD_600nm_ < 0.05), as well as the melanoma A2058 cell line with a cell survival rate of less than 50%. Fractions 1 to 4 were inactive.

### 2.2. Isolation and Identification of Cholic Acid and 3-oxo-cholic Acid

Active fraction 5 as well as inactive fractions 4 and 6 were analyzed using UHPLC-HR-MS (Figures S4 and S5). Compound **1**, with a retention time of 5.6 min, an *m*/*z* of 355.2633 [M+H]^+^ and an elemental composition of C_24_H_34_O_2_ (calc. *m*/*z* for [M-3H_2_O+H]^+^ = 355.26371), was identified as being present in considerably higher amounts in fraction 5 compared to the inactive fractions. In the ESI- mode, the *m*/*z* of compound **1** was 407.2727 [M-H]^−^, with an elemental composition of C_24_H_39_O_5_, (calc. *m*/*z* [M-H]^−^ = 407.27975), corresponding to cholic acid based on a database search using PubChem. The mass shift observed when analyzing compound **1** in the ESI- mode is indicative of the loss of 3 H_2_O molecules under ESI+. This is often observed for bile acids and sterols which undergo in-source water losses during ESI+ [17,18,19,20]. Compound **1** (1 mg) was isolated using a two-step mass-guided HPLC-fractionation approach (Figures S6 and S7), and a co-eluting compound (**2**) (0.3 mg) with an elemental composition of C_24_H_37_O_5_ and an *m*/*z* of 405.46 [M-H]^−^, which was identified as 3-oxo cholic acid based on PubChem, was also isolated.

**Figure 1:**
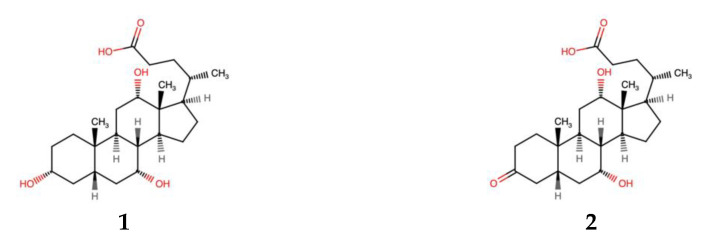
Structures of compounds **1** and **2**.

Compounds **1** and **2** were isolated as white powders. Compound **1** had the molecular formula C_24_H_40_O_5_ based on HR-IMS-MS analysis, while compound **2** had a molecular formula of C_24_H_38_O_5_. The 1D and 2D NMR data (Table 1 and Table S1) confirmed that both compounds have a C-24 steroidal structure. The ^1^H-NMR spectrum for compound **1** showed an H3 signal at 3.35 ppm, which was absent in compound **2**. The remaining NMR data for compound **1** corresponded well with those of compound **2,** accounting for the expected chemical shift perturbations caused by the oxygen in position 3. Further evaluation of the HSQC, HMBC and H2BC spectra of compound **1** was consistent with that of compound **2**, confirming their identical steroidal structures (Figure 1 and Figures S9–S18).

### 2.3. Antibacterial and Anticancer Activity of Compounds ***1*** and ***2***

Compound **1** was screened for antibacterial and cytotoxic activity and showed no activity at concentrations ranging from 5 to 100 µg/mL. It is expected that there should be a concentration-dependent activity of bile acids on cell membranes. At high concentrations, bile acids can swiftly dissolve membrane lipids (due to their detergent-like properties) and cause cell death, while lower concentrations of bile acids may affect cell membranes in a more subtle way, which does not necessarily cause cell lysis [15]. Sannasiddappa et al. [16] assessed the bioactivity of cholic acid against *S. aureus*, a common intestinal commensal. They established that cholic acid at a minimum inhibitory concentration of 20 mM, caused increased bacterial membrane disruption and the leakage of cell contents compared to using a sub-inhibitory concentration of 8 mM. This suggests that an increased concentration of cholic acid in the antibacterial assay in this study may have been required to show activity given that the concentration used in this study was only 0.245 mM.

Compound **2** was also screened for antibacterial and anticancer activity, and no activity was observed at concentrations ranging from 5 to 100 µg/mL. This result was consistent with earlier work in which Li et al. [21] isolated the same compound from a marine sponge symbiont *Psychrobacter* sp. and observed no antibacterial and anticancer activity at a concentration of 30 µg/mL. However, 3-oxo cholic acid has been shown to be a precursor in cholic acid synthesis [22]. Given that the antibacterial activity observed from cholic acid is likely concentration-dependent, and to assess whether the other *Thalassomonas* species also produce cholic acid, cholic acid was isolated from these species using analytical and preparative TLC and was confirmed by LC-MS (Figures S19–S23). The chromatographic profile showed that cholic acid (RF value of 0.35) was produced by all *Thalassomonas* species (Figure S8).

**Figure 2:**
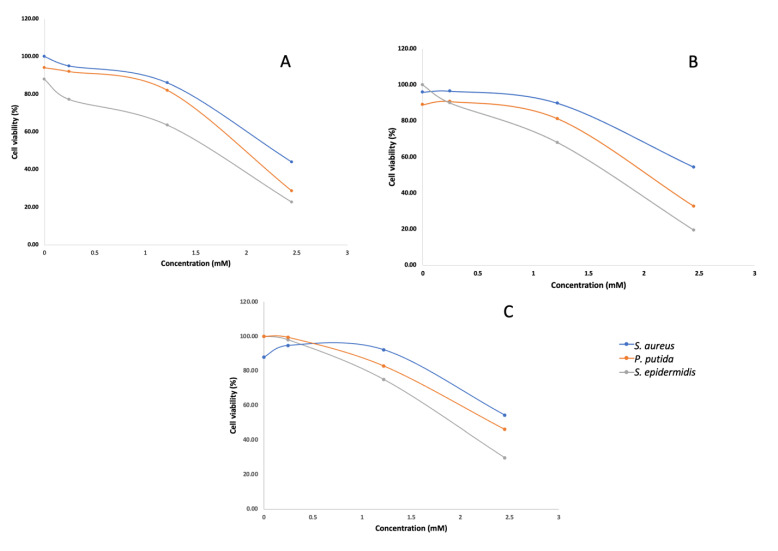
Concentration–response curves of *S. aureus*, *P. putida* and *S. epidermidis* treated with different concentrations of cholic acid isolated from *T. actiniarum* (**A**), *T. viridans* (**B**) and *T. haliotis* (**C**). Assays were conducted in triplicate with three technical repeats.

The isolated cholic acid was used in an antibacterial dose-response assay at concentrations of 100 µg/mL, 500 µg/mL and 1 mg/mL. Although antibacterial activity was observed against *S. aureus*, *S. epidermidis* and *P. putida* in the dose response assay (Figure 2), cholic acid was likely not responsible for the activity observed during the initial screening. Fraction 5 was screened at a concentration of 250 µg/mL, and the antibacterial effect was significantly greater (OD_600nm_ < 0.05) compared to that when screening cholic acid as a pure compound at a much higher concentration. It is therefore likely that the active compound was missed. Given the known antibacterial activity of cholic acid and the controversy around bile acid production in marine bacteria, we used genome mining to elucidate a biosynthetic pathway for bile acid synthesis in this genus.

### 2.4. Re-Sequencing of the Thalassomonas Genomes and Evaluation of the Genomic Capacity to Produce Cholic Acid Derivates

**Figure 3:**
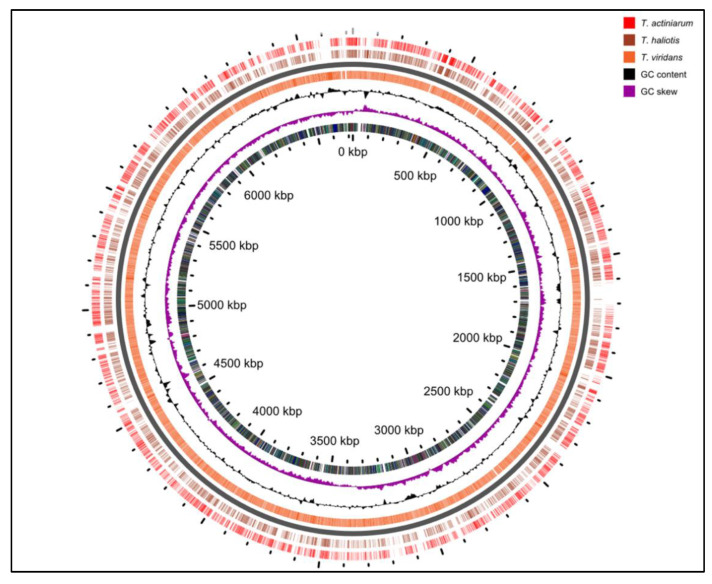
Comparison of the genomes of all three *Thalassomonas* species.

We previously reported the draft genomes for the *Thalassomonas viridans* and *T. actiniarum* species in an earlier work [23]. Our initial interest in screening *Thalassomonas* species for bioactivity stemmed from the identification of highly novel BGCs; however, many of the pathways could not be assembled as a result of the short-read technology employed. Further sequencing incorporating ONT and Illumina technologies resulted in the complete genomes of all three species, and these have been submitted to the Genbank database (Table 2). When comparing the genomes only (excluding chromids), the average nucleotide identity between the pairs of genomes was 83% between *T. viridans* and the other two species, while *T. actiniarum* had an 87% identity with *T. haliotis* (Figure 3).

These assemblies revealed that *T. viridans* and *T. actiniarum* both harbored a chromid or megaplasmid carrying the bulk of the genetic content associated with the secondary metabolism in these bacteria, which is absent in *T. haliotis*. Chromids are a feature more common to bacteria that have a symbiotic or pathogenic relationship with eukaryotes [24]. All three species were isolated from marine invertebrates [25,26]; therefore, it is interesting that *T. haliotis* lacks this feature. Secondary chromids are essential for survival in the native conditions of the bacterium but may be non-essential in other conditions, such as a laboratory environment. We have observed the spontaneous loss of the chromid in *T. viridans* through routine laboratory culturing, suggesting that these replicons may be secondary megaplasmids. Each genome carries eight copies of the operon encoding the 16S, 23S and 5S rRNA, while the chromids each carry a single such operon. The comparison of the three genomes highlighted that many of the differences in the genomes are accounted for by the presence of SM gene clusters (Figure S3). A similar comparison between the chromids at the nucleotide level indicated that although there are regions of nucleotide identity (shared gene content), they are largely unrelated (Figure S2A). The predicted BGCs identified in all three species show low or no similarity to known clusters, showcasing the potential for novel compound discovery. This includes a highly complex region spanning over 300 kb in *T. viridans*, challenging some of the largest secondary metabolite coding regions found to date (Figure S2B) [27]. When using the antiSMASH-based delineation of BGCs to calculate the portion of the genetic material dedicated to the secondary metabolism, *T. viridans*, *T. actiniarum* and *T. haliotis* dedicate 7.7%, 6.2% and 4%, respectively.

#### 2.4.1. Investigation of the Genetic Potential to Produce Bile Acids De Novo

Bile acids are water-soluble, amphipathic, steroidal molecules and are the end products of the breakdown of cholesterol in the liver of mammals and fish [28,29]. Although generally produced by eukaryotes, more and more bile acids and their derivatives have been identified in lab cultures of bacteria that are not taxonomically related, which would indicate that the production of these compounds may be more widely spread than expected [30,31,32,33]. However, recently, it was demonstrated that cholic acid isolated from marine bacteria, when cultivated in marine broth (BD Difco 2216), could be a consequence of the deconjugation of the conjugated bile acids already present in the marine broth and should not be attributed to the de novo synthesis of the compound but rather to the biotransformation of conjugated bile acids. The authors further identify putative bile salt hydrolases encoded on the genomes of *Dokdonia donghaensis*, *Maribacter dokdonensis* and *Myroides pelagicus*, which are marine bacteria reported to synthesize cholic acid de novo [34].

Notably, in addition to cholic acid, several other spots were observed in the chromatographic profile of the methanol extract of the *Thalassomonas* sp. (Figure S7), with RF values lower than that of the cholic acid standard. In the marine broth control, a smear was observed in this area of the plate, which is indicative of the sample being too concentrated. These results, together with the findings by Tueros et al., led us to investigate the genomic capacity for bile acid synthesis in this genus.

**Figure 4:**
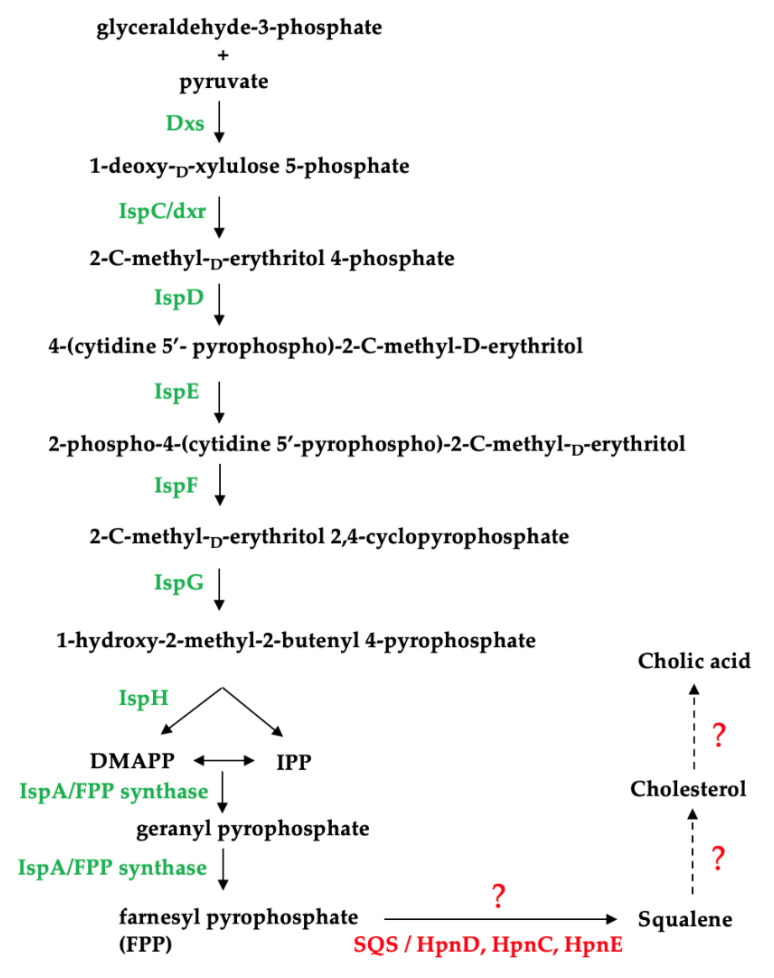
Identification of enzymes involved in the non-mevalonate pathway in *Thalassomonas* species. Dxs: 1-deoxy-D-xylulose-5-phosphate synthase; IspC: 1-deoxy-D-xylulose-5-phosphate reductoisomerase; IspD: 4-pyrophosphocytidyl-2-C-methyl-D-erythritol synthase; IspE: 4-pyrophosphocytidyl-2-C-methyl-D-erythritol kinase; IspF: 2C-methyl-D-erythritol 2,4- cyclopyrophosphate synthase; IspG: 1-hydroxy-2-methyl-2-(E)-butenyl 4-pyrophosphate synthase; IspH: 1-hydroxy-2-methyl-butenyl 4-pyrophosphate reductase and IspA: farnesyl pyrophosphate synthase; SQS: squalene synthase; HpnD and HpnC: squalene synthase; HpnE: FAD-dependent amino oxidase/phytoene desaturase.

Using the completed genomes of the *Thalassomonas* sp., we attempted to identify a biosynthetic pathway responsible for cholic acid production in this genus (Figure 4). We e established that all three species have the genetic capacity to produce the isoprenoid precursors isopentenyl pyrophosphate (IPP) and dimethylallyl pyrophosphate (DMAPP) using the non-mevalonate pathway and identified a gene encoding a putative farnesyl pyrophosphate (FPP) synthase which catalyzes the formation of FPP (Table S6), which is involved in the synthesis of the cholesterol precursor, squalene [35]. However, no squalene synthase (SQS) genes were identified based on the PROKKA annotation, which calls genes based on a threshold of 1 × 10^6^ under default parameters using a series of databases including RefSeq, UniProt, Pfam, TIGRFAMs and hmmscan [32].

The genetic basis for hopanoid synthesis in bacteria has been described and is analogous to non-mevalonate bile acid synthesis. This alternative route for squalene production in bacteria involves a three-enzyme system (HpnD, HpnC and HpnE) to synthesize squalene from FPP [36,37]. However, no HpnC, HpnD or HpnE homologs could be identified on the *Thalassomonas* sp. genomes. This would also suggest that neither hopanoids nor cholesterol can be synthesized by these strains given that squalene is a precursor for the production of both of these sterols. Squalene synthases from prokaryotes have not received as much attention as those from eukaryotes, and bifunctional squalene synthases have been characterized from bacterial strains including *Thermosynechococcus elongatus, Bacilllus megaterium* and *Methylococcus capsulatus* [38,39,40]. A protein sequence alignment of these characterized bacterial squalene synthases (*T. elongatus* Accession: BAC08649.1; *M. capsulatus* Accession: CAA71097.1, *B. megaterium* ADF40697.1) revealed that BmSQS shares 27.61% and 29.03% similarity with the squalene synthases from *T. elongatus* and *M. capsulatus*, respectively. Since the sequences are poorly conserved, it could suggest that the *Thalassomonas* species may be producing a highly novel squalene synthase.

Based on genomic analysis, the enzymes essential in cholesterol and hopanoid synthesis beyond the FPP precursor could not be identified in the *Thalassomonas* genomes, implying that this genus does not have the genetic capacity to produce bile acids de novo.

#### 2.4.2. Identification of Putative Penicillin Acylases and Potential Bile Salt Hydrolases

BSHs, also known as cholylglycine hydrolases (CGHs) or conjugated bile salt hydrolases (CBSHs), are enzymes that catalyze the hydrolysis of glycine and/or taurine-conjugated bile acids by cleaving the amide bond between the bile acid steroid nucleus and the conjugated amino acid. BSHs have mainly been identified in gut-associated bacteria from several genera including *Bifidobacteria*, *Lactobacillus*, *Enterococcus*, *Streptococcus*, *Clostridium*, *Listeria*, *Bacteriodes*, *Stenotrophomonas* and *Brucella*. It is presumed that these gut commensals use bile acid deconjugation to modify the bile acid pool, alter the gut microbiome and utilize the free amino acids released after amide hydrolysis as a source of nutrients [41,42,43,44].

BSHs are related to penicillin acylases (PAs), both structurally and phylogenetically. PAs are enzymes that hydrolyze the amide bond of beta-lactam antibiotics and are classified as penicillin G acylases (PGAs) or penicillin V acylases (PVAs), depending on the substrate preference for either benzylpenicillin or phenoxymethylpenicillin, respectively. Both PGAs and PVAs are used in industry to catalyze the production of 6-aminopenicillianic acid (6-APA), a precursor in the production of semi-synthetic antibiotics [45,46].

Although the exact role of penicillin acylases in microbial physiology is unknown, it is hypothesized that these enzymes may be involved in conferring beta-lactam antibiotic resistance to the producers, assist in acquiring alternative carbon sources or act as quorum quenchers [45,46,47].

Like BSHs, penicillin acylases belong to the Ntn-hydrolase superfamily, which includes a number of enzyme subfamilies from prokaryotes, eukaryotes and archaea, which share the N-terminal hydrolase fold and catalytic mechanism. Ntn-hydrolases have a characteristic mechanism of action which involves the nucleophilic attack of the carbonyl carbon of an amide bond by the catalytic N-terminal residues serine (PGAs) or cysteine (PVAs and BSHs) [45]. Based on a homology search against 570 representatives of the Ntn-hydrolase superfamily, we identified putative penicillin acylases from all three *Thalassomonas* sp. (Table S7), with conserved domains characteristic of Ntn-cephalosporin acylases (Ntn-CA) and Ntn-penicillin G acylase-like hydrolases (Ntn-PGA-like) (Figure S1).

The acylases were also classified as penicillin amidases (pfam01804) belonging to the N-acyl-homoserine lactone (AHL) acylase cluster of orthologs (cog2366). An amino acid sequence alignment identified the conserved residues essential for catalysis and the proteolytic cleavage of Ntn-hydrolases (Figure S25).

However, no significant sequence homology was observed between the predicted penicillin acylases from the *Thalassomonas* sp. and the CGH or PVA representatives in the database.

**Figure 5:**
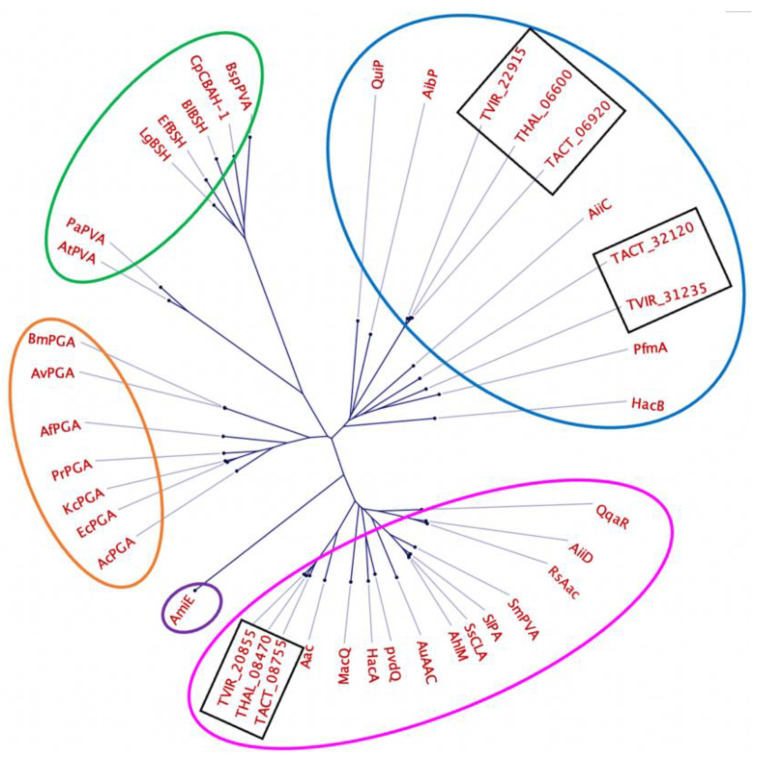
Phylogenetic tree showing the relationship between the putative penicillin acylases from *Thalassomonas* sp. (black rectangles) and members of the Ntn-hydrolase family. Circled in pink and blue are Ntn-hydrolases belonging to group A and B AHL acylases, circled in green are penicillin V acylases and bile salt hydrolases, circled in orange are penicillin G acylases and circled in purple is an amidase.

The evolutionary relationship between the predicted penicillin acylases from *Thalassomonas* and other members of the Ntn-hydrolase family was further assessed through a comparison with 32 enzymes with reported AHL acylase activity, penicillin acylase activity and bile salt hydrolase activity [48,49] (Table S8 and Figure 5).

Based on the phylogenetic analysis, the predicted penicillin acylases are very distant from enzymes with characterized BSH and penicillin V acylase activity (circled in green). The acylases TACT_32120, TVIR_31235, TVIR_22915, TACT_06920 and THAL_06600 clustered with group A acyl homoserine lactone acylases (circled in pink), while TVIR_20855, THAL_08470 and TACT_08755 clustered with group B acyl homoserine lactone acylases (circled in blue).

AHL acylases are promising antimicrobial candidates, specifically because of their ability to interfere with quorum sensing (QS). QS is a cell-density-dependent process that bacteria use to communicate by means of synthesizing AHLs that are released into the surrounding environment. These chemical signals are recognized by neighboring bacteria of the same or different species and allow for the population-wide regulation of a variety of behaviors such as antibiotic production, virulence, biofilm formation and pigment production. Since QS regulates various cellular processes involved in virulence, the application of quorum quenchers in human health, agriculture, aquaculture and biofouling is imminent [50].

While the putative acylases predicted in *Thalassomonas* do not belong to the CGH or PVA subfamilies, suggesting that these enzymes are not likely candidates involved in bile salt hydrolysis, it is important to note that the substrate scope for members of the Ntn-hydrolase superfamily varies considerably.

Furthermore, the experimentally characterized BSHs CpBSH from *Clostridium perfringens*, LgBSH from *Lactobacillus paragasseri* and JCM 5343T and PVA BsPVA from *Lysinibacillus sphaericus* are bifunctional enzymes showing both penicillin acylase and bile salt hydrolase activity, while the CGH homologs *Sl*acI and *Sl*ac2 have been shown to exhibit homoserine lactone acylase activity, but not BSH activity [45,46,51,52,53].

A large portion of the genomes of the *Thalassomonas* sp. have no functional annotation; it is therefore likely that the *Thalassomonas* species are capable of modifying sterols and bile acids with the aid of novel enzymes and perhaps pathways that have yet to be characterized. In addition, the inconsistent nomenclature used to name and define genes in different databases further complicates in silico-based analysis, necessitating the biochemical characterization of the putative penicillin acylases identified and the determination of whether they exhibit any bile salt hydrolase activity [54,55,56].

## 3. Discussion

Cholesterol synthesis by bacteria has not been demonstrated, although some bacteria are capable of using cholesterol as a carbon source, while others are capable of producing hopanoids, the cholesterol analogs found in bacterial membranes [36]. Consequently, no biosynthetic pathway to produce bile acids from cholesterol or hopanoids has been elucidated in prokaryotes, and a recent proposal that the identification of these compounds in laboratory culture supernatants is an artifact should be strongly considered.

Marine invertebrates are known to produce structurally diverse steroids [57,58,59], and many of these may be a product of the microbial transformation of the host sterols by these symbionts. In this study, we have demonstrated that the genus *Thalassomonas* has bioactive potential, as indicated by the number of potentially novel secondary metabolite gene clusters encoded on the genomes. Furthermore, we determined that *T. actiniarum* demonstrates both anti-bacterial and anti-cancer activity. In addition, we have identified putative penicillin acylases encoded on the genomes of all three *Thalassomonas* species that may be involved in bile acid transformation as well as quorum quenching. Furthermore, the completed genomes allow for future investigations into the uncharacterized pathway compounds of interest. Moreover, the biochemical characterization of the identified penicillin acylases and the role in bile salt modification and quorum quenching is necessary to validate the sequence-based functional predictions.

## 4. Materials and Methods

### 4.1. Genome Sequecning, Analysis and Mining

Genomic DNA was isolated using the Macherey-Nagel Nucleospin Microbial DNA isolation kit (REF 740235.50). From the genomic DNA, two sequencing libraries were prepared per strain, one for sequencing on the MiSeq plastform (Illumina Inc.,San Diego, CA , USA) and one for sequencing on the MinION platform (Oxford Nanopore Technologies, Oxford, UK). The former consisted of a TruSeq DNA PCR-free library which was run in a 2 × 300 nt run using a 600-cycle MiSeq reagent kit v3 (Illumina Inc.). For ONT sequencing, the ligation-based sequencing kit SQK-LSK109 was used to prepare the library, which was in turn run on an R9.4.1. flow cell. Base-calling of the raw data was performed with guppy v3.4.5. The canu assembler v1.8 [60] was used to assemble the ONT data, resulting in one contig per replicon. These were subsequently polished using, firstly, the ONT data and the medaka polisher v0.11.5 [61] and, secondly, the Illumina data and the Pilon polisher v1.22 [62] for a total of 10 rounds. For the first five, bwa mem [63] was used as a mapper; for the final five, Bowtie2 [64] was applied. In addition, the Illumina data were assembled into 25–98 scaffolds containing 69–163 contigs using Newbler v2.8. Both assemblies were combined and manually curated using Consed [65], resulting in the complete genomes. Annotation was performed using PROKKA v1.11 [32]. The annotated genomes were deposited at GenBank under the accession numbers CP05963, CP059733, CP059734, CP059735 and CP059736. The complete genomes of *T. actiniarum*, *T. viridans* and *T. haliotis* were assessed for secondary metabolite biosynthesis gene clusters using antiSMASH v6.0.1 [66], with all optional features on. Using NCBI, the sequences of 570 representatives of the Ntn-hydrolase superfamily (cd01901, downloaded on 16 September 2022) were extracted and used as a database to search for homologs of the Ntn hydrolase superfamily in the complete genome sequences of each *Thalassomonas* sp. Hits with an E. value of 0.0 were considered as putative members of this enzyme class. Conserved domains were determined using BLASTp on NCBI, and sequence alignments were constructed using the CLC Genomics Workbench 22 (Qiagen) (for the phylogenetic tree) and Clustal Omega [67] for the alignment of the AHL acylases. The phylogenetic tree was constructed using the neighbor-joining method and a bootstrap of 1000 replicates.

### 4.2. Cultivation and Extraction of Secondary Metabolites from T. actiniarum

*T. actiniarum* was cultivated in 6 × 1 L conical flasks at 28 °C at 150 rpm in 500 mL Marine Broth (Difco) for 14 days. Metabolites secreted into the medium were extracted using 40 g/L Diaion^®®^HP-20 resin beads (Sigma-Aldrich, St. Louis, MO, USA). The beads were prepared by soaking in 100% MeOH (Sigma-Aldrich) for 20 min and were washed extensively with Milli-Q water prior to adding them to the cultures 3–4 days before extraction. The separation of the beads from the culture was performed under vacuum, and the cultures were filtered through a fine mesh cheesecloth filter (Dansk Hjemmerproduktion, Denmark) using a filtration flask and Büchner funnel. Beads captured on the cheesecloth filter were washed with 100 mL of Milli-Q water and separated from the water, as described previously. The beads were extracted twice with 150 mL 100% MeOH, followed by vacuum filtration of the MeOH extract through a Whatman^®®^ 90 mm No. 3 filter (Sigma-Aldrich). The extract was then dried under reduced pressure and stored at −20 °C. For the TLC purification of the cholic acids from *T. actiniarum*, *T. haliotis* and *T. viridans*, the strains were cultivated in 4 × 1 L conical flasks, and extraction was performed as described above. The methanol extract was concentrated by evaporation in a fume hood to a final volume of 10 mL and stored at 4 °C

### 4.3. Flash Liquid Chromatography Fractionation of the Extract from T. actiniarum

Diaion^®®^HP-20ss resin beads (6.5 g) were prepared as described above and packed in a flash cartridge (Biotage, Uppsala, Sweden) prior to equilibration in 5% MeOH. The dried extract was dissolved in 8 mL 90% MeOH in water, and 2 g of Diaion^®®^HP-20ss resin beads was added before drying the mixture under reduced pressure. The resin/extract mixture was loaded on top of the packed cartridge. Fractionation was performed using a Biotage SP4™ system (Biotage, Sweden) with flow rate of 12 mL/min and a gradient of 5–100% MeOH over 32 min and MeOH:acetone to 100% acetone over 18 min. This resulted in 21 fractions that were pooled to 6 fractions and dried under reduced pressure at 40 °C. The stock solution of the fractions was prepared in DMSO to a final concentration of 40 mg/mL and diluted with Milli-Q water to a working stock of 1 mg/mL for bioactivity assays.

### 4.4. Isolation and Purification of Cholic Acids from Thalassomonas sp. Using Thin-Layer Chromatography

The methanol extracts were separated using TLC conducted on a 20 × 20 cm aluminum plate coated with silica gel 60 G F_254_ with a thickness of 165–235 µm Merck, JHB, SA. Cholic acid, cholesterol and deoxycholic acid standards (Sigma-Aldrich) and the extracts were applied at a concentration of 0.25 µg/µL, and 2 µL of each methanol fraction was applied. Marine broth was used as a control and prepared in the same way as the extracts. The samples were applied 2 cm from the bottom of the plate, and the development distance was 10 cm from the start. The mobile phase used was *n*-hexane-ethyl acetate-methanol-acetic acid in a volume composition of 20:20:5:2 in a final volume of 50 mL. After development, the plate was dried in a fume hood at room temperature. For visualization, the TLC plate was sprayed generously with a solution of 10% phosphomolybdic acid in ethanol (Sigma-Aldrich) and dried with a heat gun until spots appeared. The remaining extract was developed on a 20 × 20 cm glass preparative TLC Silica gel 60 G F254 plate with a layer thickness of 250 µm (Merck) and was applied as horizontal streaks along the breadth of the plate, 2 cm from the bottom. The samples were developed as described above, and a portion of the plate was stained to determine where the spots of interest were located. Spots of interest were scraped off using a sterile surgical blade and extracted with 5 mL 100% MeOH overnight. The methanol extract was dried to completion in a fume hood, resuspended in DMSO to a final concentration of 40 mg/mL and stored at room temperature.

### 4.5. Bioactivity Screening

#### 4.5.1. Growth Inhibition Assay

The bacterial strains used as test organisms and the corresponding culturing conditions are depicted in Table 3. Strains marked with an asterisk were maintained on blood agar (BD Difco) and used for the screening of flash fractions and pure compounds isolated from *T. actiniarum*. Unmarked strains were maintained on Lysogeny agar and were used for the antibacterial screening of cholic acids isolated from the *Thalassomonas* sp. Briefly, fresh colonies were inoculated into the appropriate medium and incubated overnight at 37 °C with shaking at 150 rpm. Following overnight incubation, 2 mL of cell suspension was inoculated into 25 mL of fresh media and incubated at 37 °C for their respective incubation periods (Table 3), with shaking at 150 rpm. Following incubation, cultures were diluted in the appropriate culture media to the desired bacterial density (Table 3). Diluted culture samples were added to a 96-well microtiter plate to a total volume of 50 µL/well. Flash fractions and pure compounds (50 µL) were screened in triplicate at concentrations of 250 µg/mL and 5–100 µg/mL, respectively. For the concentration–response assay, cholic acid was tested in triplicate at concentrations of 100 µg/mL, 500 µg/mL and 1 mg/mL. Plates were incubated overnight at 37 °C without shaking before measuring the growth at an absorbance of 600 nm using a Spectrostar Nano plate reader. The bacterial suspension diluted with water was used as a growth control. A dilution series of gentamycin from 32 to 0.01 µg/mL was used as a positive control in the assays, and uninoculated growth medium was used as a negative growth control. Extracts were considered active if the absorbance at 600 nm was less than 0.05. Antibacterial assays were performed in triplicate with three technical repeats.

#### 4.5.2. Cytotoxicity Assay

The cell viability of fractions and pure compounds **1** and **2** was tested using an in vitro cell proliferation assay against the melanoma cell line A2058. The pure compound was tested for cytotoxicity against the human colon cancer cell line HT29 and the normal lung fibroblast cell line MRC5. The cancer cell lines A2058 and HT-29 were cultivated in Dulbecco’s Modified Eagle’s Medium (D-MEM) with 10% Fetal Bovine Serum (FBS, Biochrom, Cambridge, UK), 0.01 mg/mL gentamycin and 1% L-alanyl-L-glutamine (Biochrom, UK) and Roswell Park Memorial Institute medium (RPMI-1640, Biochrom, UK), respectively, at 37 °C with 5% CO_2_ for 24 h in a Sanyo CO_2_ incubator. The non-malignant MRC-5 cells used as toxicity controls were cultivated in Minimum Essential Medium with Earle’s salts (E-MEM, Biochrom, UK) media with 10% FBS, 0.01 µg/mL gentamycin, 1% L-alanyl-L-glutamine, 1% non-essential amino acids (NEAA, Biochrom, UK), 1% sodium pyruvate (Biochrom, UK) and 1% sodium bicarbonate (Biochrom, UK) and incubated under the same conditions as the cancer cell lines. The cells were split to keep them in a monolayer. To split the cells, the culture medium was discarded, and the cells were washed with Dulbecco’s PBS (Sigma) for 1 min. After washing the cells, 0.25% trypsin (in PBS) was added to the cells and swirled gently for 10 s. The trypsin solution was discarded, and the flask was incubated at 37 °C for 5 min. Once detached from the culture flask, the cells were resuspended in 10 mL of fresh growth media. An appropriate volume of this suspension (depending on cell density) was added to 15 mL fresh medium and incubated at 37 °C with 5% CO_2_ for 24–48 h. Following incubation, cells were counted using a haemocytometer, and cell morphology and density were assessed using a microscope. For the A2058 cell line, a density of 2000 cells per well is required for the assay, while the HT-29 and MRC-5 (toxicity control) cell lines are required at a cell density of 2000 and 4000 cells/well, respectively. For screening, 100 µL of the cell suspension (at the desired cell density) was seeded into 96-well tissue culture plates. The seeded plates were incubated for 24 h at 37 °C with 5% CO_2_. Following 24 h of incubation and prior to adding the flash fractions to the wells, the medium was removed from the wells and replaced with fresh RPMI-1640, the assay media. Flash fractions (prepared in Section 4.3) were added to the wells at a final concentration of 100 µg/mL and tested in triplicate, while pure compounds were screened at concentrations of 5 µg/mL, 25 µg/mL, 50 µg/mL and 100 µg/mL. The plates were incubated for 72 h at 37 °C with 5% CO_2_.

After incubation, 10 µL of Aqueous One solution (Promega) was added to each well, and the plates were incubated again for 1 h at 37 °C with 5% CO_2_. After incubation, the absorbance was measured at 485 nm using a DTX 880 Multimode Detector and the Multimode Analysis Software v4.5 (Beckman Coulter, CA, USA). Cell survival was calculated using the following equation: Survival % = (AbsF-AbsP) × 100/(AbsN–AbsP). Fractions inhibiting cell survival by 50% were considered active.

To calculate cell survival in the MTS proliferation assay, AbsF is the average absorbance at 485 nm of wells with fractions, AbsP is the average absorbance at 485 nm of wells with the positive control and AbsN is the average absorbance at 485 nm of wells with the negative control.

### 4.6. Dereplication, Isolation and Structure Elucidation of Compounds ***1*** and ***2*** from T. actiniarum

High-resolution mass spectrometry was run with positive and negative electrospray ionization using UPLC-MS. This was performed on an Acquity UPLC I-class coupled with a Vion IMS QToF (Waters). For chromatographic separation, an Acquity UPLC C18 column (1.7 um, 2.1 mm × 100 mm) (Waters) was used. The samples were run with a 12 min gradient increasing from 10% to 90% acetonitrile (Sigma-Aldrich), with 0.1% FA (Sigma-Aldrich) in Milli-Q ultra-pure water and a flowrate 0.45 mL/min; the column temperature was set to 40 °C. To process the data, Waters UNIFI 1.9.4 Scientific Information System Software was used.

A two-step mass-guided HPLC fractionation approach was used for compound purification and performed on a prep-HPLC system (Waters) consisting of a 600 HPLC pump, a Waters flow splitter for a flow rate of 2–8 mL/min and a split ratio of 100:1, a 3100 mass spectrometer, a 2996 photo diode array detector and a 2767 sample manager. The system was controlled with MassLynx version 4.1. Various columns were used (all from Waters): Xterra RP-18 Prep Column (10 um, 10 mm × 250 mm) and XSelect CSH Prep Fluorophenyl (5 um, 10 mm × 250 mm). Gradients were optimized using Milli-Q water with 0.1% FA (Sigma-Aldrich) and acetonitrile (Sigma-Aldrich) with 0.1% FA as the mobile phase. The flow rate was constant at 6 mL/min. Flash fraction 5 was resuspended in 100% MeOH, and the initial separation was carried out on the Xterra RP-18 preparative column, followed by a second round of isolation using the XSelect CSH Prep Fluorophenyl column with gradient compositions, as described in the Supplementary Materials (Tables S2 and S3).

#### 4.6.1. Identification of Cholic Acid Isolated from *Thalassomonas* Strains Using LC-MS and LC-MS/MS

Cholic acid isolated from the *Thalassomonas* species was identified using LC-MS with an API 4000 triple Quadrupole mass spectrometer (Sciex, Redwood City, CA USA) coupled with a Dionex Ultimate 3000 micro-HPLC system and set in negative ionization mode. The multi-step gradient (Table S4) was generated at 300 µL/min using Milli-Q water with 0.1% FA (solvent A) and MeOH (Burdick & Jackson) with 0.1% FA (solvent B). Separation was achieved on an Agilent Poroshell C18 column (2.1 mm × 50 mm × 2.7 μm). A cholic acid standard was used to determine the retention time of cholic acid in the samples. LC-MS/MS analysis of cholic acid isolated from *T. actiniarum* only was conducted with a Q-Exactive quadrupole-Orbitrap mass spectrometer (Thermo Fisher Scientific) coupled with a Dionex Ultimate 3000 nano-UPLC system and set in negative ionization mode. Data were acquired using XCalibur v4.1.31.9, Chromeleon v6.8, Orbitrap MS v2.9 and Thermo Foundations 3.1 and set to acquire data for *m*/*z* 407.28. Samples were loaded on a C18 trap column (300 μm × 5 mm × 5 μm), trapped onto the column and washed for 3 min prior to eluting onto the analytical column. Chromatographic separation was performed with a Waters nanoEase (Zenfit™, MA, USA M/Z Peptide CSH C18 column (75 μm × 25 cm × 1.7 μm). The solvent system employed was LC water (Burdick and Jackson BJLC365, Muskegon, MI, USA) and 0.1% FA (solvent A) and acetonitrile and 0.1% FA (solvent B). All data were acquired using Proxeon stainless steel emitters (Thermo Fisher Scientific).

The multi-step gradient was generated at 300 nL/min, as described in Table S5. LC-MS analysis was conducted by the Centre for Proteomic and Genomic Research.

#### 4.6.2. Structural Elucidation of Compounds **1** and **2** from *T. actiniarum*

All NMR spectra were acquired on a Bruker Avance III HD spectrometer equipped with an inverse detected TCI probe with cryogenic enhancement on ^1^H, ^2^H and ^13^C, operating at 599.90 MHz and 150.86 MHz for ^1^H and ^13^C, respectively. Samples were prepared in acetonitrile-*d*_3_ and methano-d4 and recorded at 298 K. All experiments were acquired using standard pulse sequences for Proton, Carbon, DQF-COSY, HSQC, HMBC, H2BC, HSQC-TOCSY (mlev), TOCSY (dipsi2) and NOESY in Topspin 3.5pl7, using gradient selection and adiabatic inversion, where applicable, and processed in Mnova 12.0.0. Spectra were referenced on the residual solvent peak acetrontrilel-*d_3_* (δH = 1.94 and δC = 1.32).

## Figures and Tables

**Table 1:** ^1^H and ^13^C-NMR data for cholic acid (1) in acetonitrile-d_3_.

Cholic Acid (1)			
Position	δ_C_, Type	δ_H_ (յ in Hz)	δ_OH_ (յ in Hz)
1	35.98, CH2	0.90 (d, *J* = 6.5 Hz, 3H),	
2	30.62, CH2	1.32 (ddd, *J* = 15.9, 9.8, 6.4 Hz, 1H)	
3	72.22, CH	3.32 (tt, *J* = 11.2, 4.4 Hz, 1H)	3.35
4	39.78, CH2	2.05 (td, *J* = 13.2, 11.6 Hz, 1H)	
5	42.34, CH	1.88 (ddd, *J* = 14.6, 5.6, 3.4 Hz, 1H)	
6	35.44, CH2	1.43 (dt, *J* = 14.8, 2.2 Hz, 1H)	
7	68.85, CH	3.74 (q, *J* = 3.0 Hz, 1H)	3.77
8	40.23, C		
9	27.26, CH	2.09 (ddt, *J* = 35.0, 17.8, 5.6 Hz, 1H)	
10	35.18, C		
11	28.81, CH2	1.52 (tdd, *J* = 12.4, 10.1, 2.9 Hz, 3H)	
12	73.58, CH	3.90 (t, *J* = 3.0 Hz, 1H)	3.92
13	47.05, C		
14	42.48, CH	1.25 (tdd, *J* = 14.6, 5.6, 3.4 Hz, 1H)	
15	23.87, CH2	1.03 (qd, *J* = 12.0, 6.1 Hz, 1H)	
16	28.19, CH2	1.23 (tdd, *J* = 12.4, 10.1, 2.9 Hz, 3H)	
17	47.52, CH	1.72 (q, *J* = 7.4 Hz)	
18	22.86, CH3	0.84 (s, 3H)	
19	12.81, CH3	0.63 (s, 3H)	
20	17.56, CH3	0.92 (d, *J* = 6.5 Hz, 3H)	
21	36.17, CH2	1.37 (q, *J* = 7.1 Hz)	
22	31.76, CH2	1.31 (tdd, *J* = 12.4, 9.8, 2.6 Hz, 3H)	
23	31.68, CH2	2.31 (ddd, *J* = 15.5, 10.2, 5.1 Hz, 1H)	
24	177.87, C		

**Table 2:** Genome sequencing statistics for *Thalassomonas* species.

Bacterium/Chromid	Genome/Chromid Size bp	Coverage Illumina	Coverage ONT	G + C%	Number of ORFs	tRNAs	rRNA
*T. viridans*	6,726,747	129.1	107.2	49.2	5793	119	25
pTvir	1,218,661			47.6	937	-	3
*T. actiniarum*	6,556,942	117.4	87.3	47.7	5592	116	25
pTact	937,696			46.4	682	2	3
*T. haliotis*	6,450,155	155.1	82.7	47.4	5418	116	25

**Table 3:** Test bacteria, their growth media and their incubation periods to reach the desired 0.5 McFarland standard.

Bacterial Strain	Growth Media	Incubation Period (h)	Desired Bacterial Density
*S. aureus ATCC 25923	Mueller Hinton	2.5	0.5–3 × 10^5^ CFU/mL
*E. coli ATCC 25922	Mueller Hinton	1.5	0.5–3 × 10^5^ CFU/mL
*E. faecalis ATCC 29122	Brain Heart Infusion	1.5	0.5–3 × 10^5^ CFU/mL
*P. aeruginosa ATCC 27853	Mueller Hinton	2.5	3–7 × 10^4^ CFU/mL
*Streptococcus a ATCC 12386	Brain Heart Infusion	1.5	0.5–3 × 10^5^ CFU/mL
*S. epidermidis ATCC 14990*	Lysogeny Broth	2.5	0.5–3 × 10^5^ CFU/mL
*P. putida KT2440*	Lysogeny Broth	2.5	0.5–3 × 10^5^ CFU/mL
*S. aureus ATCC 29213*	Lysogeny Broth	2.5	0.5–3 × 10^5^ CFU/mL

Strains marked with an asterisk were maintained on blood agar (BD Difco) and used for the screening of flash fractions and pure compounds isolated from *T. actiniarum*. Unmarked strains were maintained on Lysogeny agar and were used for the antibacterial screening of cholic acids isolated from the *Thalassomonas* sp.

## Data Availability

The data that support the findings of this study are available from the corresponding authors upon reasonable request.

## Supplementary Materials

The following are available online at https://www.mdpi.com/article/10.3390/md21010002/s1, Figure S1: Enzyme subfamilies belonging to the Ntn-hydrolase superfamily (NCBI), Figure S2A,B: S2A: BLASTn-based comparison between pTact and pTvir chromids, Figure S2B: 300kb BGC located on the pTvir chromid, Figure S3: Count of various biosynthetic gene cluster types on the chromosomes and chromids of the *Thalassomonas* species predicted by antiSMASH v6.0.1, Figure S4: BPI chromatogram of active fraction 5 and inactive fractions 4 and 6 in ESI+ mode, Figure S5: BPI chromatogram of active fraction 5 and inactive fractions 4 and 6 in ESI- mode, Figure S6: BPI chromatogram of the initial isolation of compound 1 from fraction 5 separated over a the Xterra RP C18 column using the mass as fraction triggers, Figure S7: BPI chromatogram of the second isolation of compound 1 (dimer 815.8) and the co-eluting compound 2 (dimer 811.7) separated over an RP fluorophenyl HPLC column using the mass as a fraction trigger, Figure S8: TLC chromatogram of the methanol extracts of *T. actiniarum* (TaCA), *T. viridans* (TvCA) and *T. haliotis* (ThCA) developed alongside a cholic acid standard (0.1 µg), a cholesterol standard (0.1 µg) and a deoxycholic acid standard (0.5 µg). Marine broth was used as a control and prepared in the same way. Figure S9–S14: 1D Proton, 1D Carbon, HSQC-HMBC, HSQC-H2BC, HSQC-TOCSY, NOESY and ROESY spectra for compound 1, S15–S18: 1D Proton, 1D Carbon, HSQC-HMBC and HSQC-H2BC spectra for compound 2, Figure S19: Standard curve of the cholic acid standard (1000 pg) with a retention time of 5.20 min, Figure S20–S22: Retention time chromatograms of cholic acid isolated from *Thalassomonas* sp., Figure S23: Comparison of the full-scan (*m*/*z* 50–435) negative-ion mass spectrum of cholic acid (standard) and cholic acid isolated from *T. actiniarum*, Figure S24: Amino acid sequence alignment of putative penicillin acylases from *Thalassomonas* sp. with AHL acylase homologues, Table S1: ^1^H and ^13^C-NMR data for 3-oxo cholic acid in acetonitrile-*d_3_*, Table S2: Mobile phase gradient used for the Xterra RP C18 column in the first round of purification, Table S3: Mobile phase gradient used for the X-Select Fluoro-phenyl column in the second round of purification, Table S4: Mobile phase gradient used for the Agilent Poroshell C18 column, Table S5: Mobile phase multi-step gradient used for the Waters nanoEase (Zenfit) M/Z Peptide CSH C18 column, Table S6: Enzymes involved in the production of the isoprenoid precursors IPP and DMAPP using the non-mevalonate pathway identified on the genomes of the *Thalassomonas* sp., Table S7: Putative penicillin acylases identified on the genomes of the *Thalassomonas* sp., Table S8: Name, accession number and descriptions of enzymes from the Ntn-hydrolase superfamily used for the construction of the phylogenetic tree.
