# Supplementary material for: Bioassay-Guided Fractionation Leads to the Detection of Cholic Acid Generated by the Rare Thalassomonas sp
Source: Mar Drugs. 2022 Dec 20;21(1):2. doi: 10.3390/md21010002 (PMC9860883; doi:10.3390/md21010002)
Supplement: Supplementary file 1 [file marinedrugs-21-00002-s001.zip › marinedrugs-2024350-supplementary (2).pdf]

marine drugs

Supplementary Material: Bioassay-Guided Fractionation Leads to the Detection of Cholic Acid Generated by the Rare *Thalassomonas* sp.

Table S1: ^{1}H and ^{13}C-NMR data for 3-oxo cholic acid in acetonitrile-d_3

3-oxo Cholic acid (2)			
**Position**	**δ_C, Type**	**δ_H, (J in Hz)**	**δ_{OH} (J in Hz)**
1	36.51, CH2	1.37 (td, *J* = 13.2, 11.2 Hz, 3H)	
2	36.52, CH2	2.10 (ddd, *J* = 15.9, 9.8, 6.4 Hz, 1H)	
3	212.05, C		
4	45.36, CH2	2.07 (dt, *J* = 14.2, 5.6, 3.4 Hz,1H)	
5	43.33, CH	1.75 (ddd, *J* = 14.2, 5.2, 3.0 Hz, 1H)	
6	33.70, CH2	1.52 (dt, *J* = 14.6, 2.2 Hz, 1H)	
7	67.46, CH	3.81 (t, *J* = 3.0 Hz, 1H)	3.77
8	39.55, C		
9	27.01, CH	2.33 (ddt, *J* = 35.0, 17.8, 5.6 Hz, 1H)	
10	34.73, C		
11	28.70, CH2	1.62 (tdd, *J* = 12.4, 10.1, 2.9 Hz, 3H)	
12	72.05, CH	3.97 (t, *J* = 3.0 Hz, 1H)	3.94
13	46.26, C		
14	41.71, CH	1.87 ((tdd, *J* = 14.6, 5.6, 3.4 Hz, 1H)	
15	22.83CH2	1.10 (qd, *J* = 12.0, 6.1 Hz, 1H)	
16	27.24, CH2	1.32 (tdd, *J* = 12.2, 10.2, 3.4 Hz, 3H)	
17	46.72, CH	1.78 (d, J=6.5)	
18	21.12, CH3	1.01 (s, 3H)	
19	11.92, CH3	0.54 (s, 3H)	
20	16.60,CH3	0.59 (d, *J* = 6.5 Hz, 3H)	
21	35.20, CH2	1.42 (tdd, *J* = 12.4, 9.8, 2.6 Hz, 3H)	
22	30.82, CH2	1.34 (ddd, *J* = 15.5, 10.2, 5.1 Hz, 1H)	
23	30.27, CH2	2.23 (ddd, *J* = 15.5, 10.2, 5.1 Hz, 1H),	
24	174.61, C		

Table S2: Mobile phase gradient used for Xterra RP C18 column in the first round of purification

Time (minutes)	Flowrate (ml/minutes)	Mobile phase A %	Mobile phase B %
0	6	80	20
2	6	80	20
30	6	0	100
40	6	0	100

Table S3: Mobile phase gradient used for X-Select Fluoro-phenyl column in the second round of purification

Time (minutes)	Flowrate (ml/minutes)	Mobile phase A %	Mobile phase B %

0	6	75	25
10	6	0	100
25	6	0	100

Table S4: Mobile phase gradient used for Agilent Poroshell C18 column

Time (minutes)	Flowrate (ml/minutes)	Mobile phase A %	Mobile phase B %
0	0.3	70	30
0.30	0.3	70	30
3	0.3	10	90
4	0.3	10	90
4.1	0.3	70	30
8.6	0.3	70	30

Table S5: Mobile phase multi-step gradient used for Waters nanoEase (Zenfit) M/Z Peptide CSH C18 column

Time (minutes)	Flowrate (ml/minutes)	Mobile phase A %	Mobile phase B %
0.00	0.3	95	5
4.00	0.3	95	5
24.00	0.3	10	90
34.00	0.3	10	90
34.10	0.3	95	5
49.10	0.3	95	5

Table S6: Enzymes involved in the production of the isoprenoid precursors IPP and DMAPP using the non-mevalonate pathway identified on the genomes of the *Thalassomonas* sp.

Enzyme	*T. actiniarum*	*T. viridans*	*T. haliotis*
**dxs**	TACT_05675	TVIR_06100	THAL_05520
**ispA**	TACT_05680	TVIR_06105	THAL_05525
**ispB**	TACT_02500	TVIR_27275	THAL_02435
**ispC/dxr**	TACT_20775	TVIR_08630	THAL_19605
**ispD**	TACT_05175	TVIR_05590	THAL_05060
**ispE**	TACT_20975	TVIR_08425	THAL_19810
**ispF**	TACT_05180	TVIR_05595	THAL_05065
**ispG**	TACT_04395	TVIR_04815	THAL_04310
**ispH**	TACT_22380	TVIR_06575	THAL_21275

Table S7: Name, accession number and descriptions of enzymes from the Ntn-hydrolase superfamily used for construction of the phylogenetic tree.

Name	Accession number	Description
PfmA	ASS36259.1	AHL acylase, partial [Pseudoalteromonas flavipulchra JG1]
AiiC	BAB75623.1	all3924 [Nostoc sp. PCC 7120 = FACHB-418]
HacB	AAY39885.1	Penicillin amidase [Pseudomonas syringae pv. syringae B728a]
QuiP	AAG04421.1	QuiP [Pseudomonas aeruginosa PAO1]
EcPGA	sp|P06875.2|	PAC_ECOLX Penicillin G acylase subunit alpha
KcPGA	sp|P07941.1|	PAC_KLUCR Penicillin G acylase subunit alpha
PrPGA	AAP86197.1	penicillin G amidase [Providencia rettgeri]
AcPGA	AAY25991.1	penicillin G acylase [Achromobacter sp. CCM 4824]
AfPGA	AAB71221.1	penicillin G acylase precursor [Alcaligenes faecalis]
BmPGA	sp|Q60136.1|	PAC_PRIMG Penicillin G acylase subunit alpha
AvPGA	sp|P31956.1|	PAC_RHIVS Penicillin G acylase subunit alpha
AibP	AAL53453.1	penicillin acylase [Brucella melitensis bv. 1 str. 16M]
AiiD	AAO41113.1	acy-homoserine lactone acylase [Ralstonia sp. XJ12B]
Aac	WP_011002462.1	MULTISPECIES: penicillin acylase family protein [Ralstonia]
QqaR	WP_010889514.1	N-acyl homoserine lactone acylase QqaR [Deinococcus radiodurans]
MacQ	BAV56778.1	N-Acyl-L-homoserine lactone acylase [Acidovorax sp. MR-S7]
pvdQ	AAG05773.1	3-oxo-C12-homoserine lactone acylase PvdQ [Pseudomonas aeruginosa PAO1]
HacA	AAY37014.1	Peptidase S45, penicillin amidase [Pseudomonas syringae pv. syringae B728a]
AhlM	AAT68473.1	N-acylhomoserine lactone-degrading acylase [Streptomyces sp. M664]
SsCLA	BAD07025.1	cyclic lipopeptide acylase [Streptomyces sp. FERM BP-5809]
SlPA	AAU09670.1	penicillin V acylase precursor [Streptomyces lavendulae subsp. lavendulae]
SmPVA	BAF51977.1	penicillin V acylase [Streptomyces mobaraensis]
AuAAC	WP_043523659.1	acylase [Actinoplanes utahensis]
Aac	BAF94155.1	acy-homoserine lactone acylase [Shewanella sp. MIB015]
AmiE	BAP18758.1	acylhomoserine lactone acylase [Acinetobacter sp. Ooi24]
AtPVA	pdb|5J9R|	C Chain C, Choloylglycine hydrolase
PaPVA	pdb|4WL2|	H Chain H, Putative exported choloylglycine hydrolase
EfBSH	pdb|4WL3|	D Chain D, Bile salt hydrolase
LgBSH	WP_020806888	MULTIPSECIES: cholylglycine hydrolase
CpCBAH-1	P54965	B chain B. bile salt hydrolase
BlBSH	pdb|2HF0|	B Chain B, Bile salt hydrolase
BspPVA	pdb|3PVA|	H Chain H, Protein (penicillin V Acylase)
THAL_08470		*T. haliotis*, predicted AHL acylase
TACT_08755		*T. actiniarum*, predicted AHL acylase
TVIR_20855		*T. viridans*, predicted AHL acylase
THAL_06600		*T. haliotis*, predicted AHL acylase
TACT_06920		*T. actiniarum*, predicted AHL acylase

TVIR_22915	*T. viridans*, predicted AHL acylase
TVIR_31235	*T. viridans*, predicted AHL acylase
TACT_32120	*T. actiniarum*, predicted AHL acylase

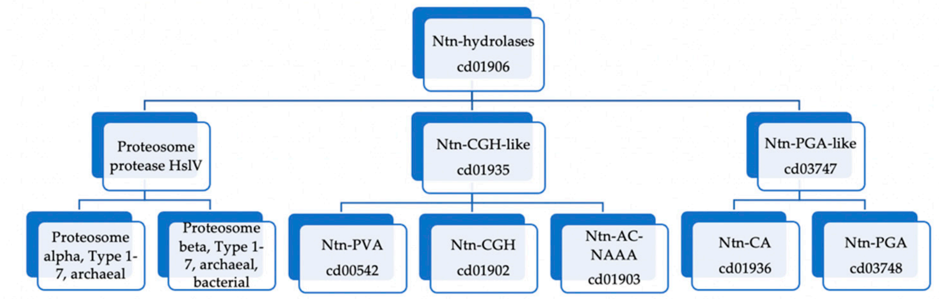

Figure S1: Enzyme Subfamilies belonging to the Ntn- hydrolase superfamily (NCBI).

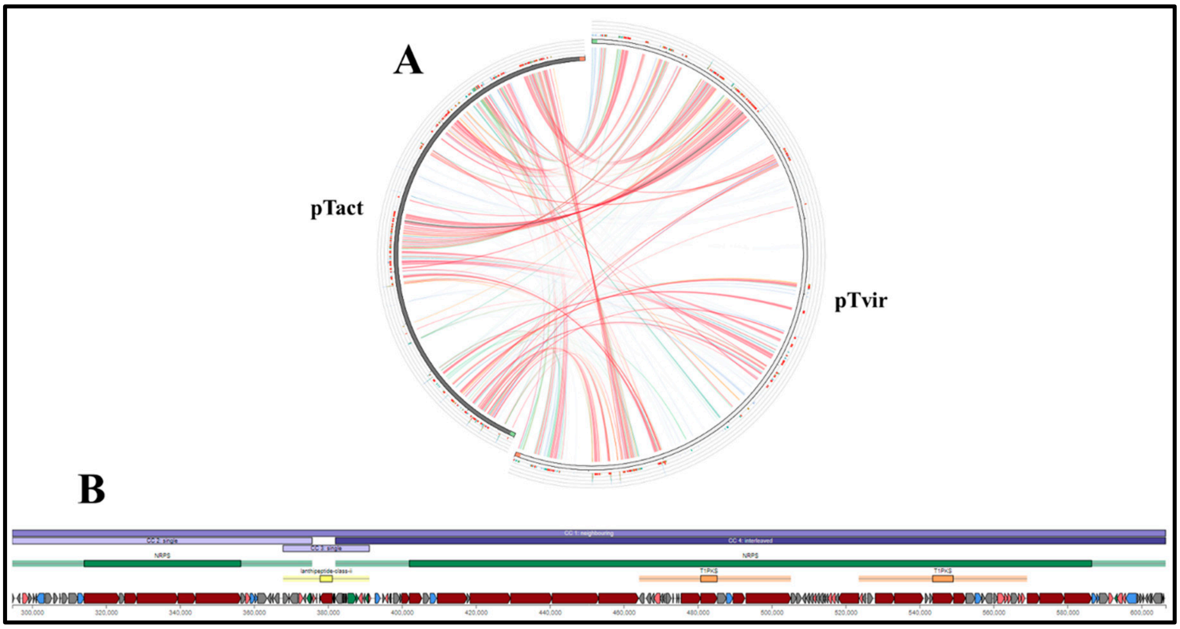

Figure S2: A) BLASTn based comparison between pTact and pTvir chromids. Ribbons are coloured according to E-value. B) 300kB BGC located on pTvir chromid.

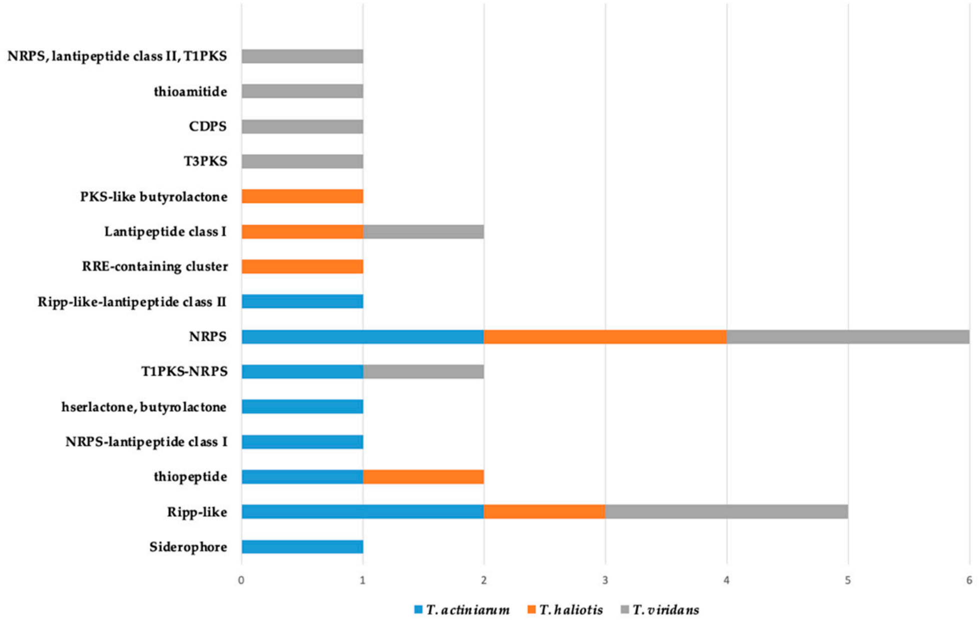

Figure S3: Count of various biosynthetic gene cluster types on the chromosomes and chromids of the *Thalassomonas* species predicted by antiSMASH v6.0.1.

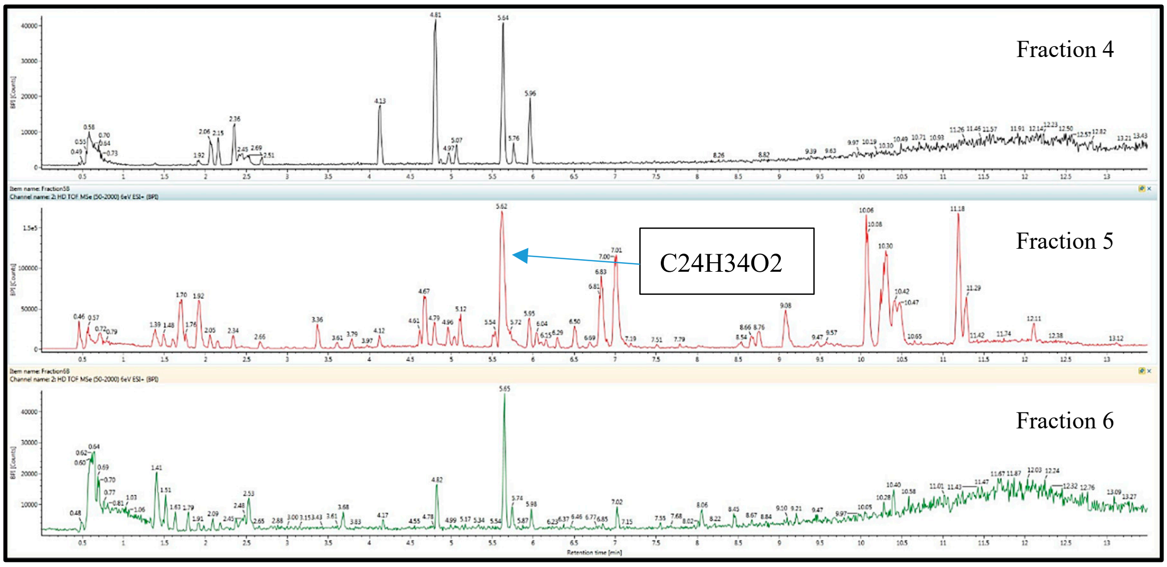

Figure S4: BPI chromatogram of active fraction 5 and inactive fractions 4 and 6 analysed in positive ionization using UHPLC-HR-MS. Compound 1 with elemental composition C24H34O2 was significantly more abundant in the active fraction that the inactive fraction.

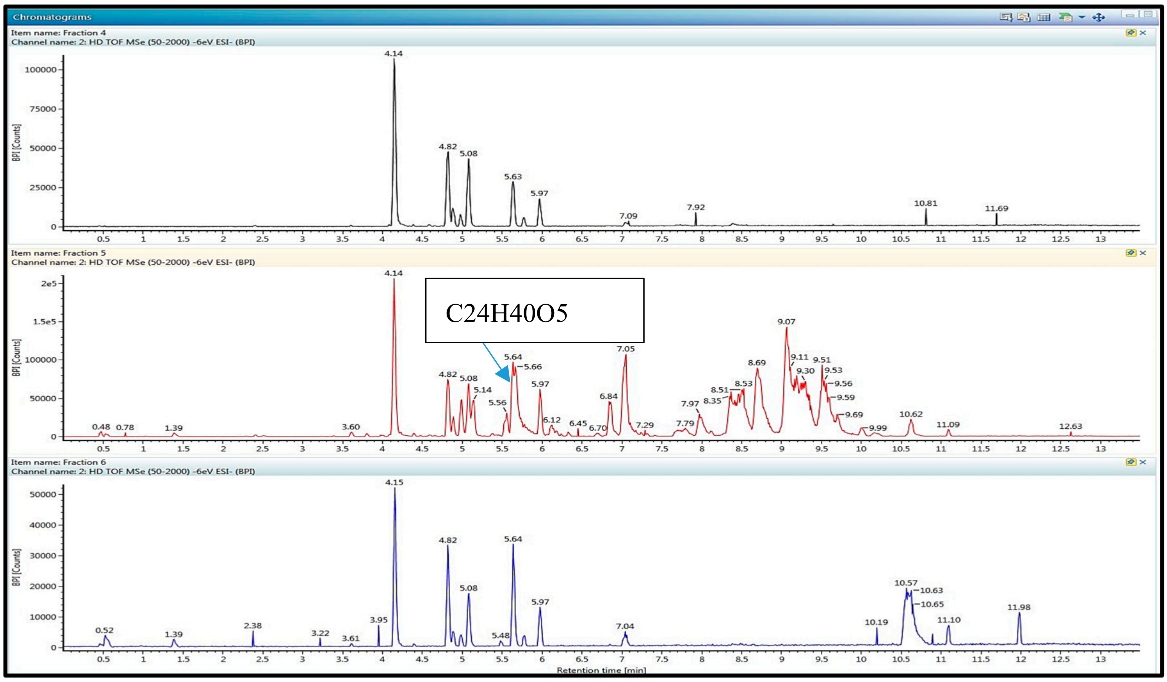

Figure S5: BPI chromatogram of active fraction 5 and inactive fractions 4 and 6 analysed in negative ionization using UHPLC-HR-MS showing compound 1 with elemental composition C24H40O5.

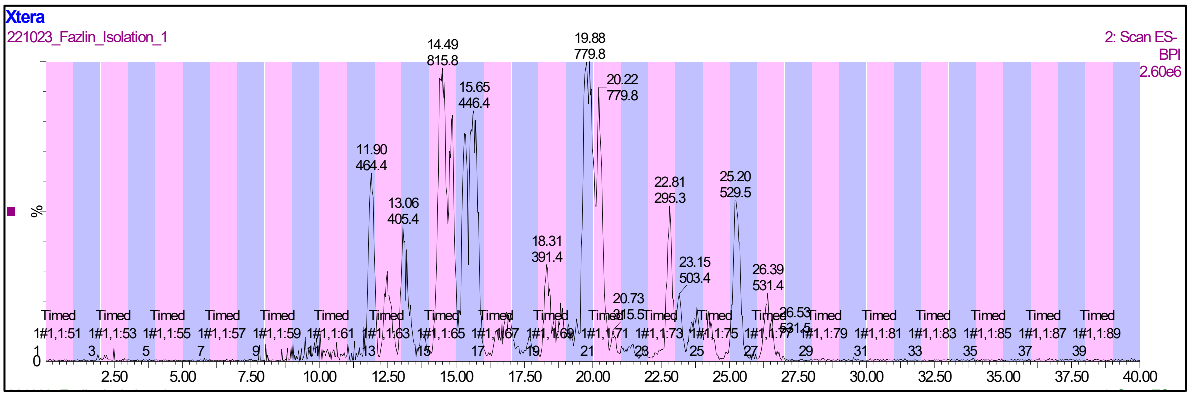

Figure S6: BPI chromatogram of the initial isolation of compound 1 from Fraction 5 separated over an Xterra RP C18 column using mass as fraction triggers.

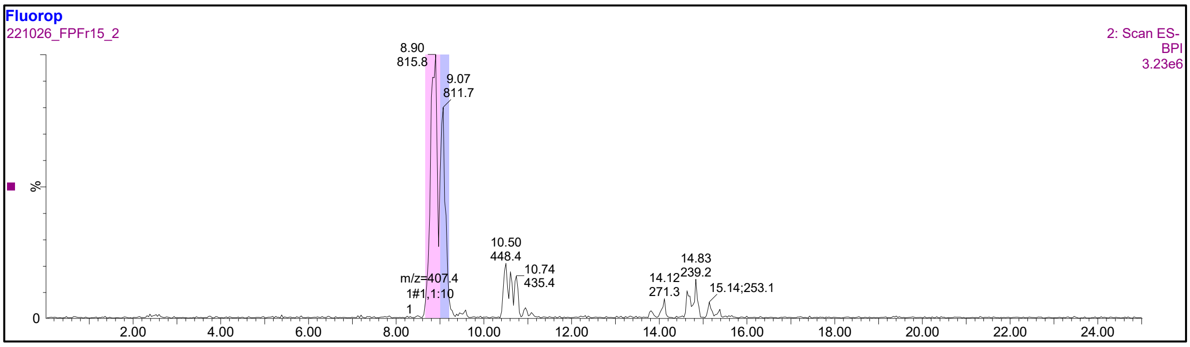

Figure S7: BPI chromatogram of the second isolation of compound 1 (dimer 815.8) and the co-eluting compound 2 (dimer 811.7) separated over a RP fluorophenyl HPLC column using mass as a fraction trigger.

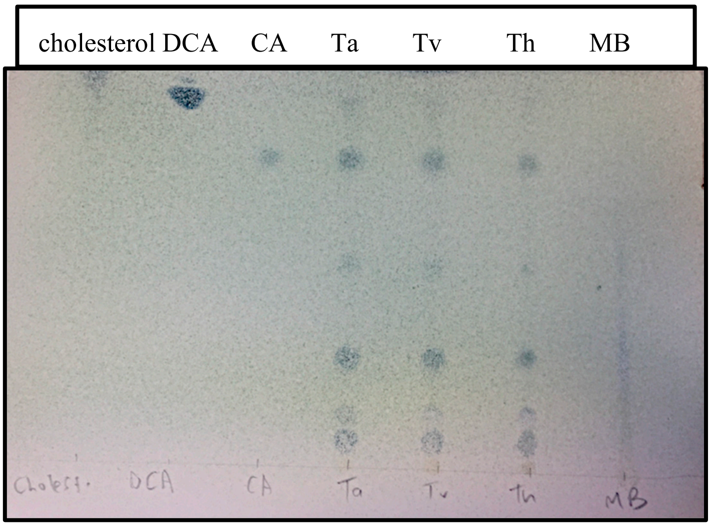

Figure S8: TLC chromatogram of the methanol extract of *T. actiniarum* (Ta), *T. viridans* (Tv) and *T. haliotis* (Th) developed alongside a cholic acid standard (0.1µg), cholesterol standard (0.1 µg) and a deoxycholic acid (DCA) standard (0.5 µg). Marine broth was used as a control and prepared in the same way as the methanol extract.

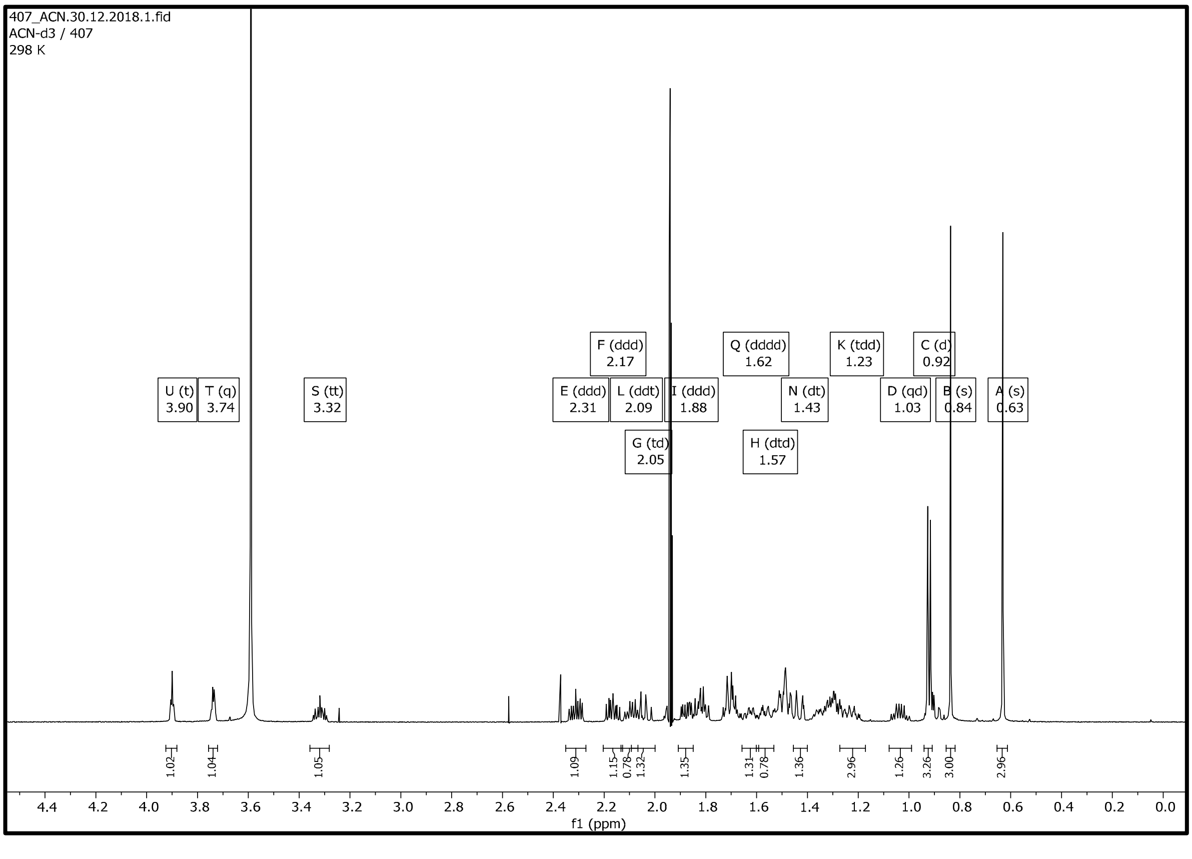

Figure S9: 1D Proton NMR spectrum for compound 1

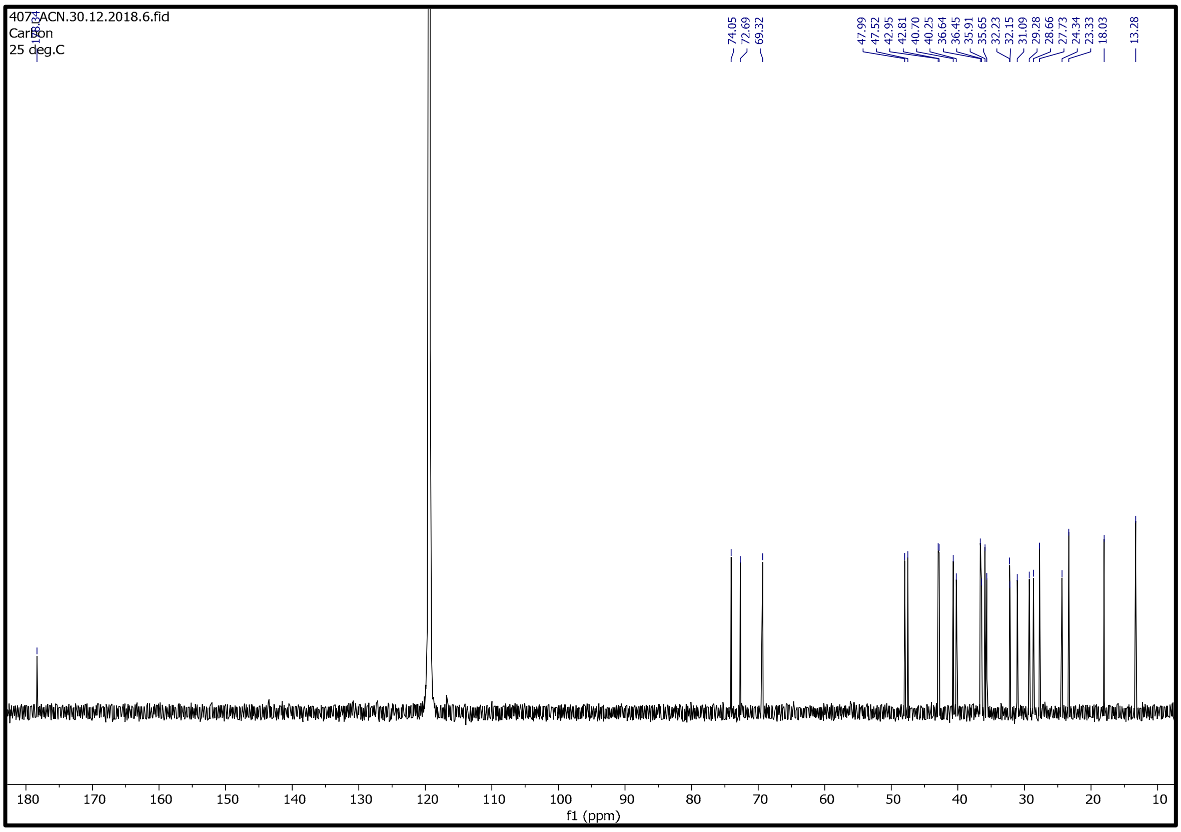

Figure S10: 1D Carbon NMR spectrum for compound 1

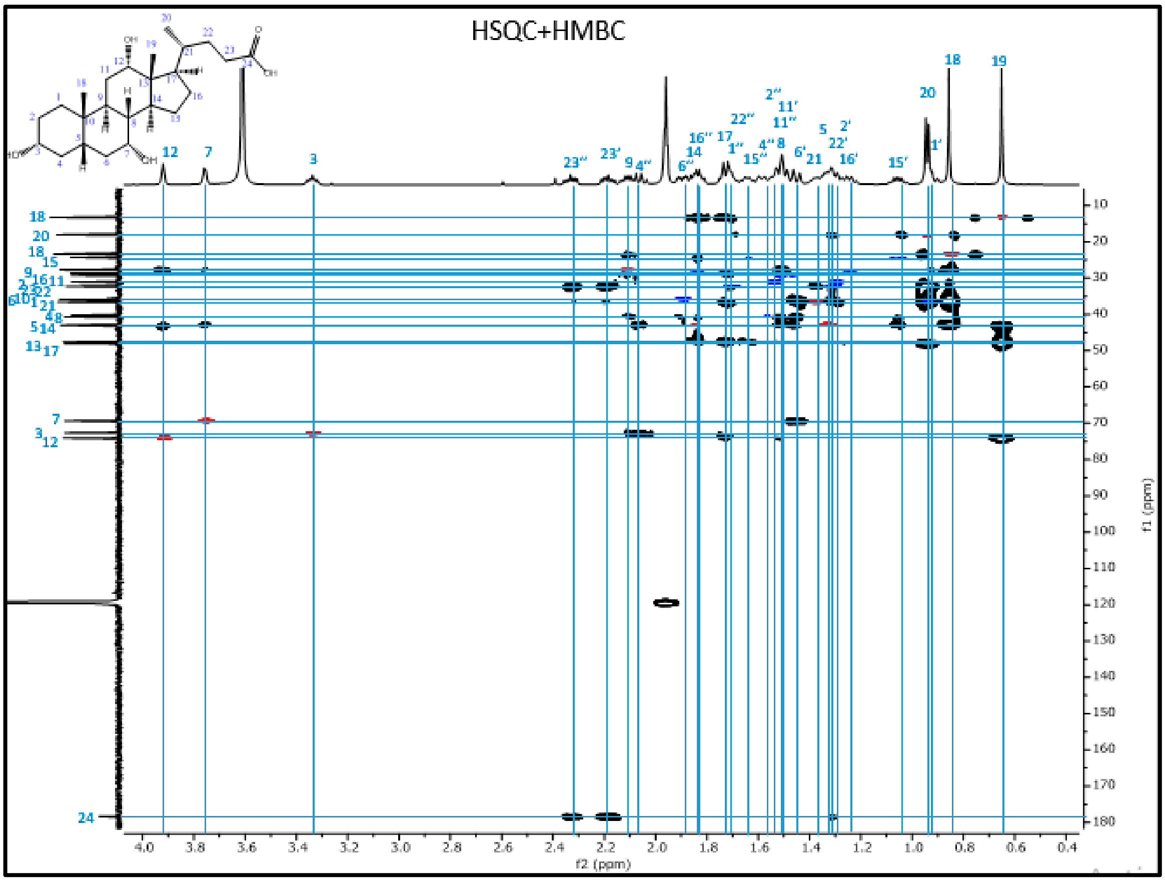

Figure S11: HSQC (red) and HMBC (blue+black) spectrum of compound 1

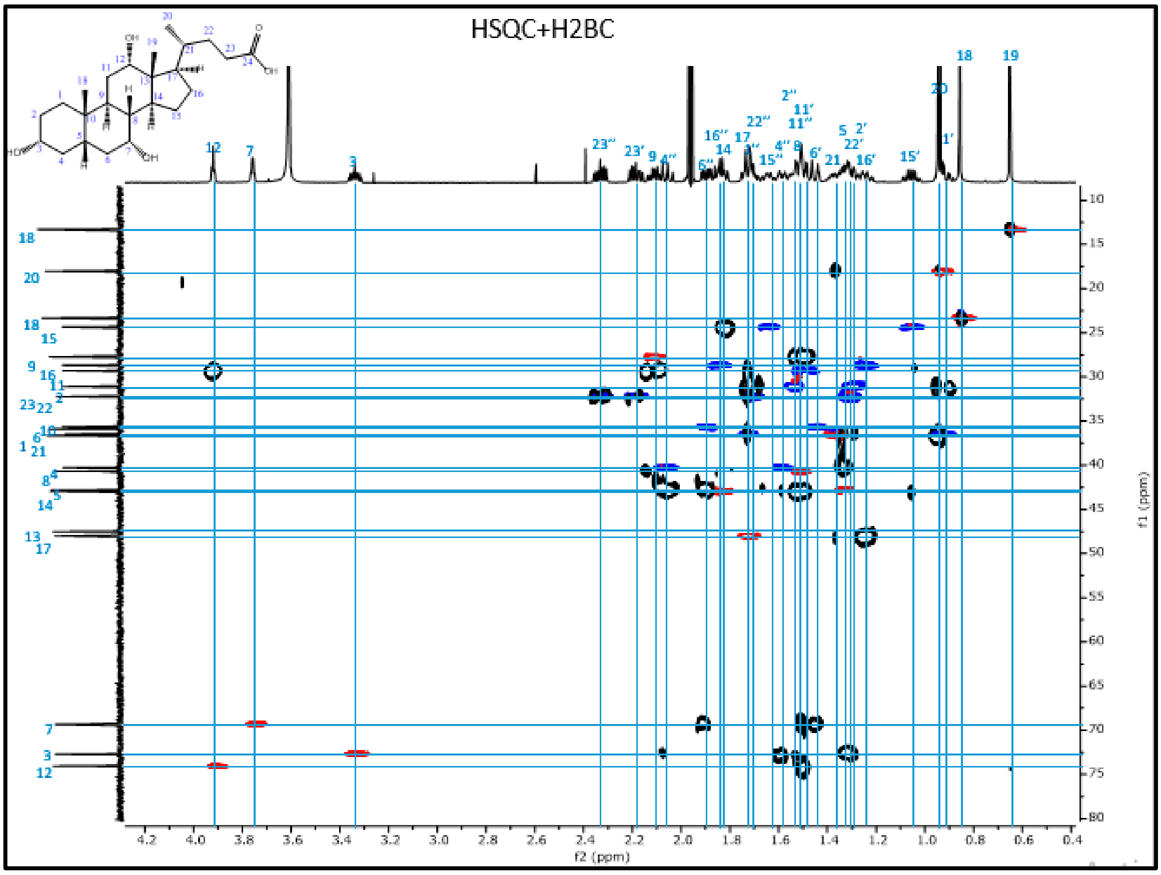

Figure S12: HSQC (red) and H2BC (blue+black) spectrum of compound 1

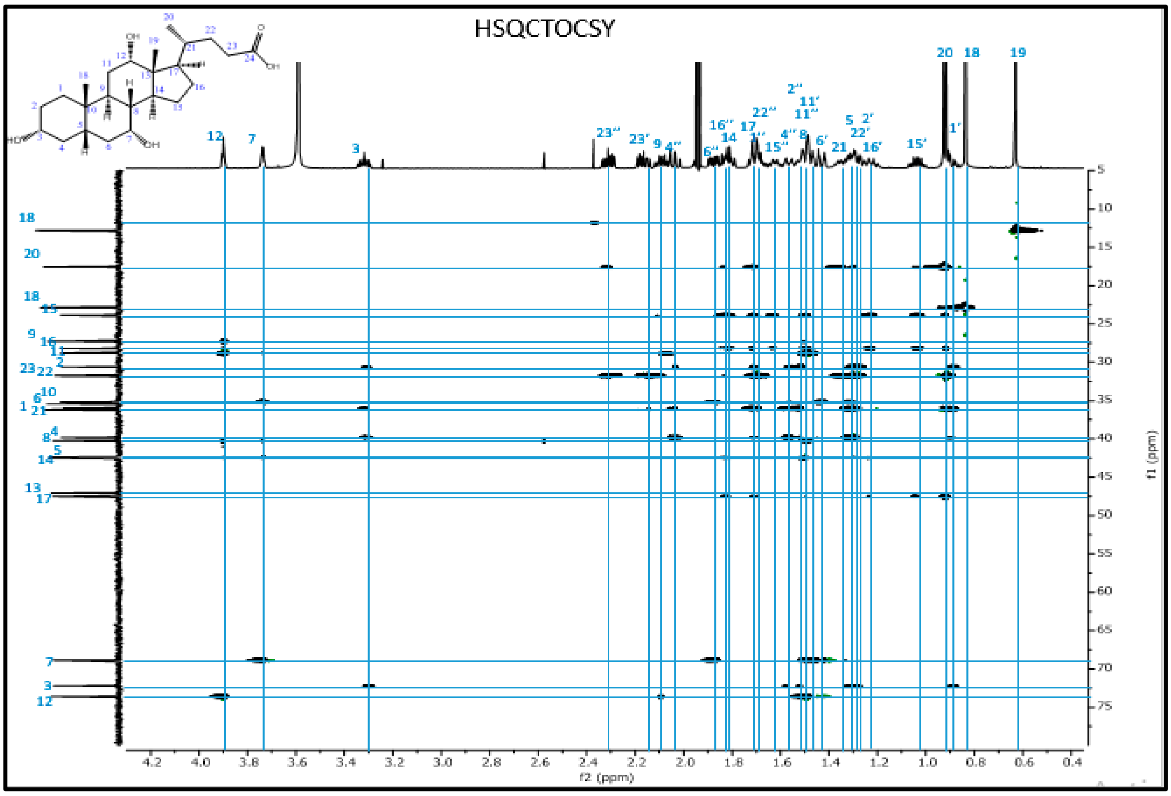

Figure S13: HSQC-TOCSY spectrum of compound 1

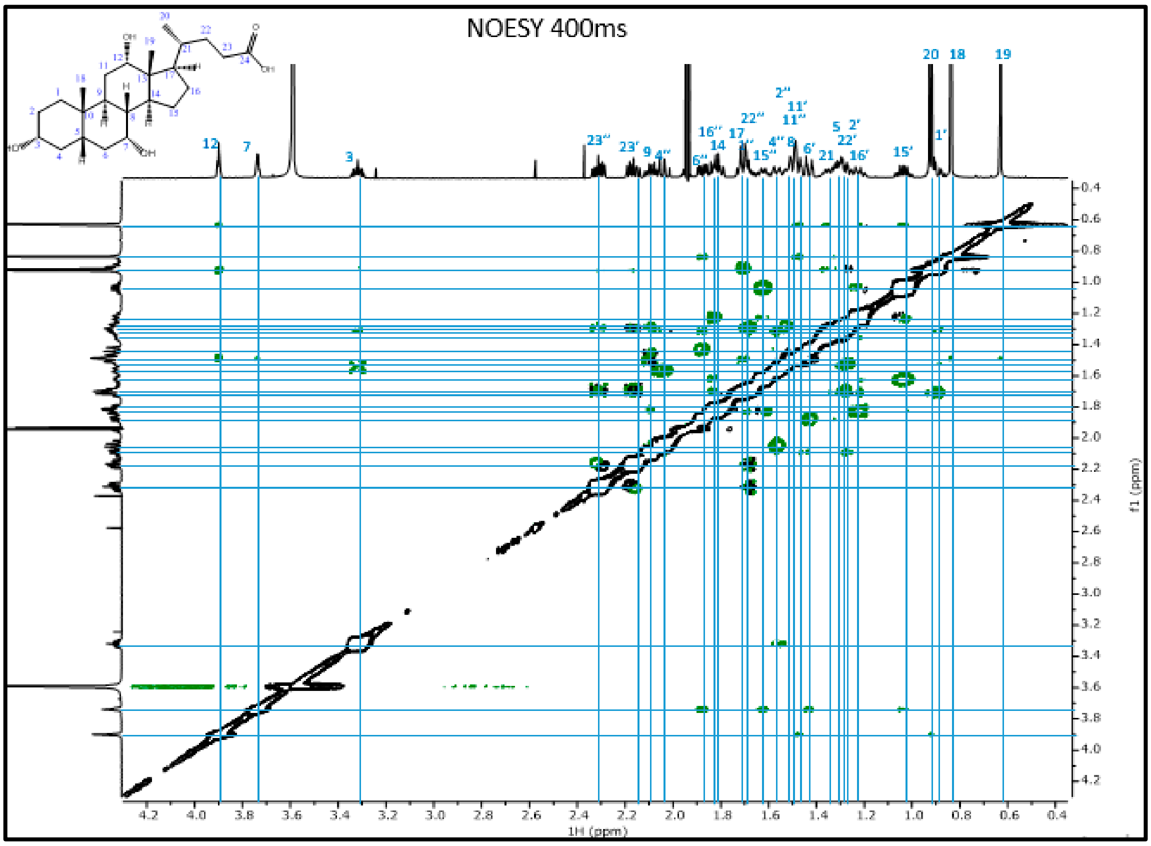

Figure S14: NOESY (400 ms mixing time) spectrum of compound 1

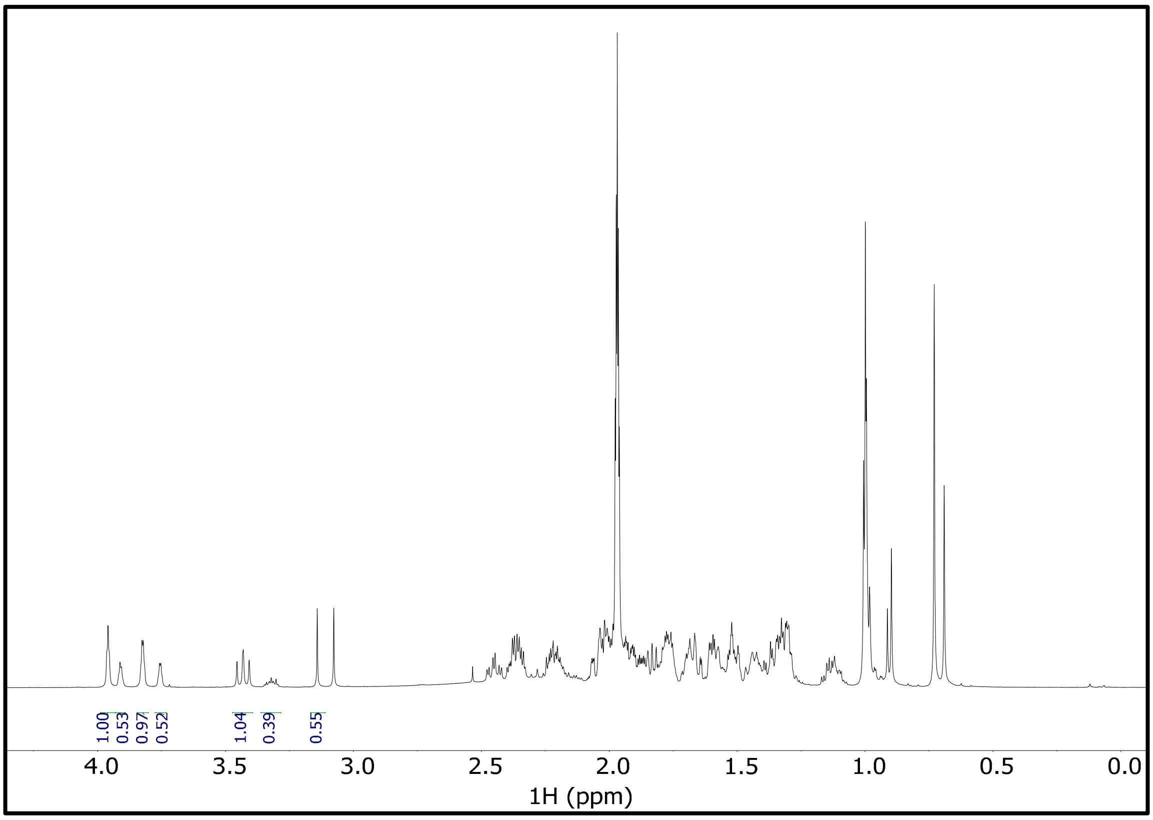

Figure S15: 1D Proton NMR spectrum for compound 2

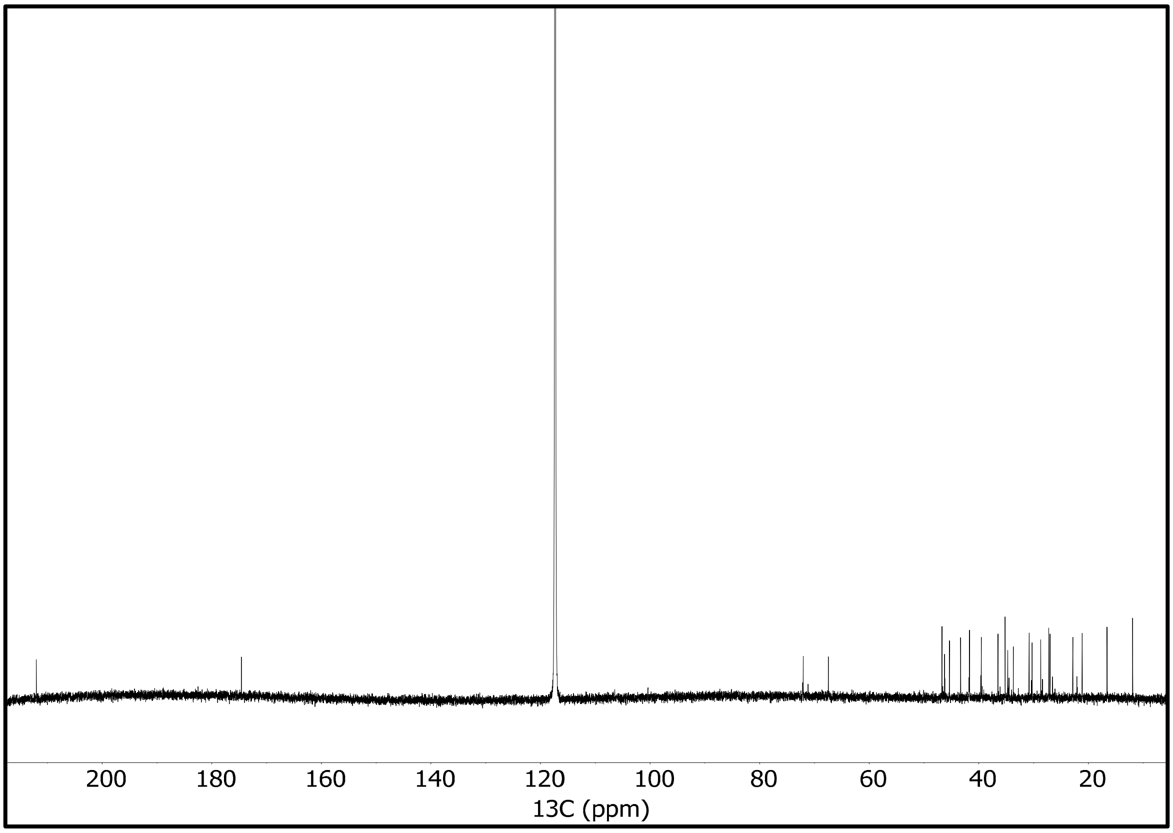

Figure S16: 1D Carbon NMR spectrum of compound 2

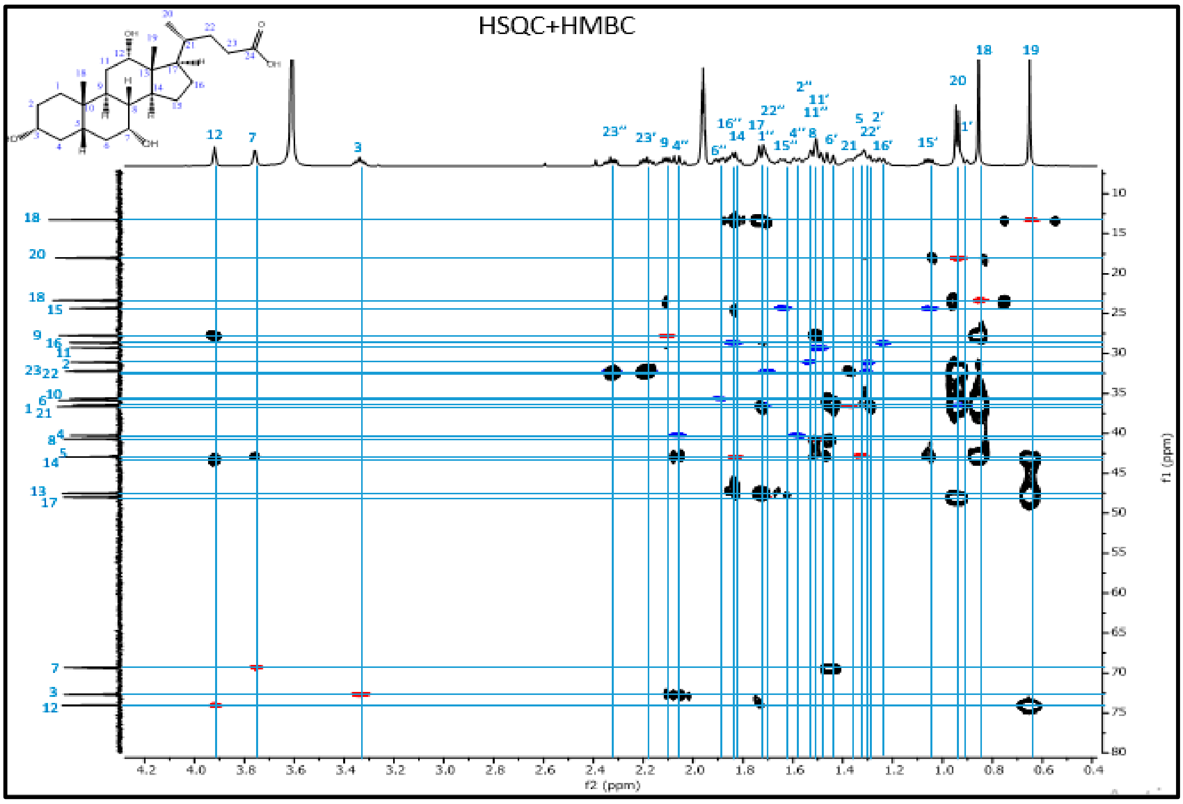

Figure S17: HSQC-HMBC spectrum of compound 2

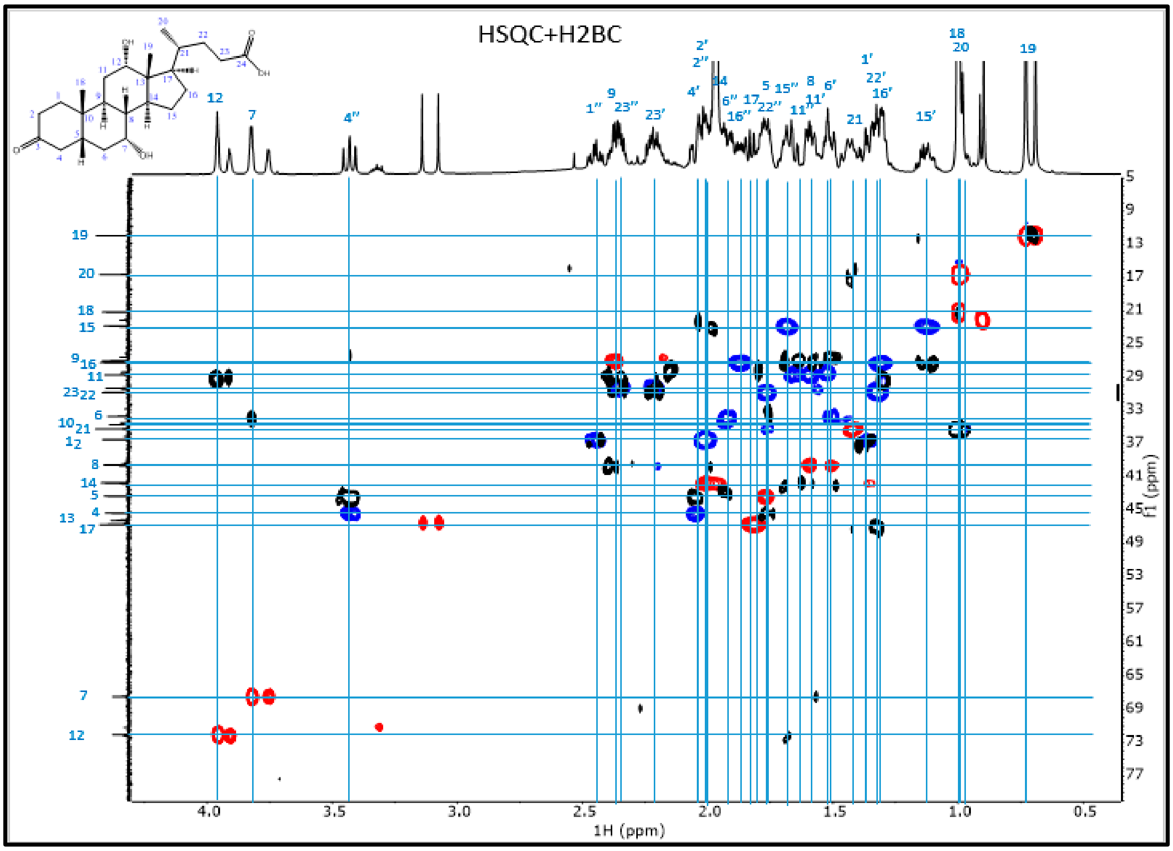

Figure S18: HSQC (red) and H2BC (blue+black) spectrum of compound 2

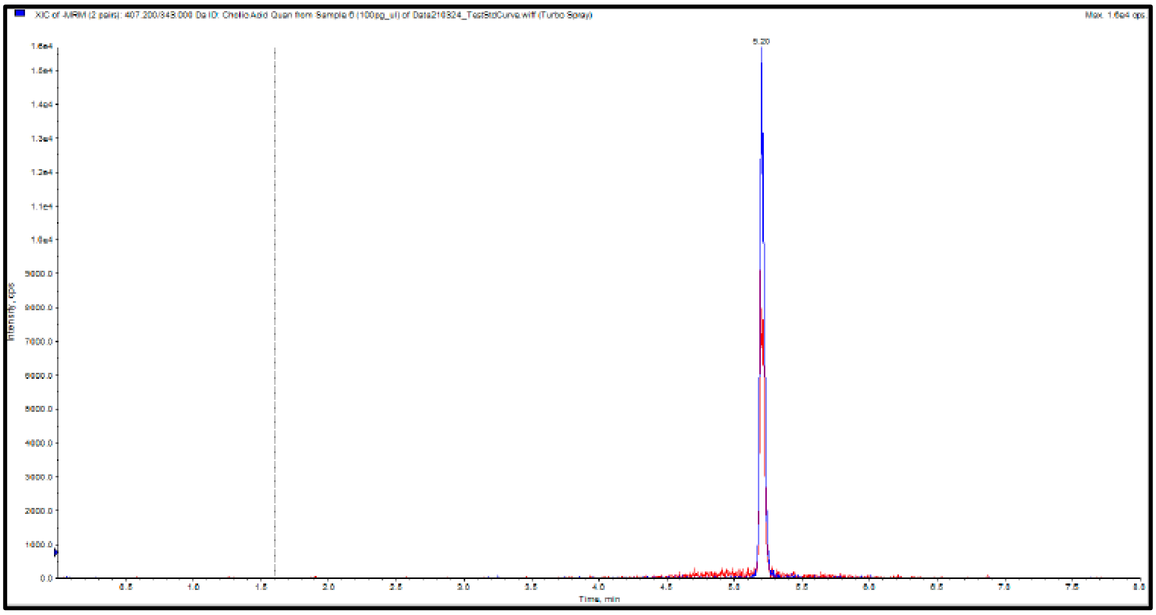

Figure S19: Standard curve of the cholic acid standard (1000pg) with a retention time of 5.20mins.

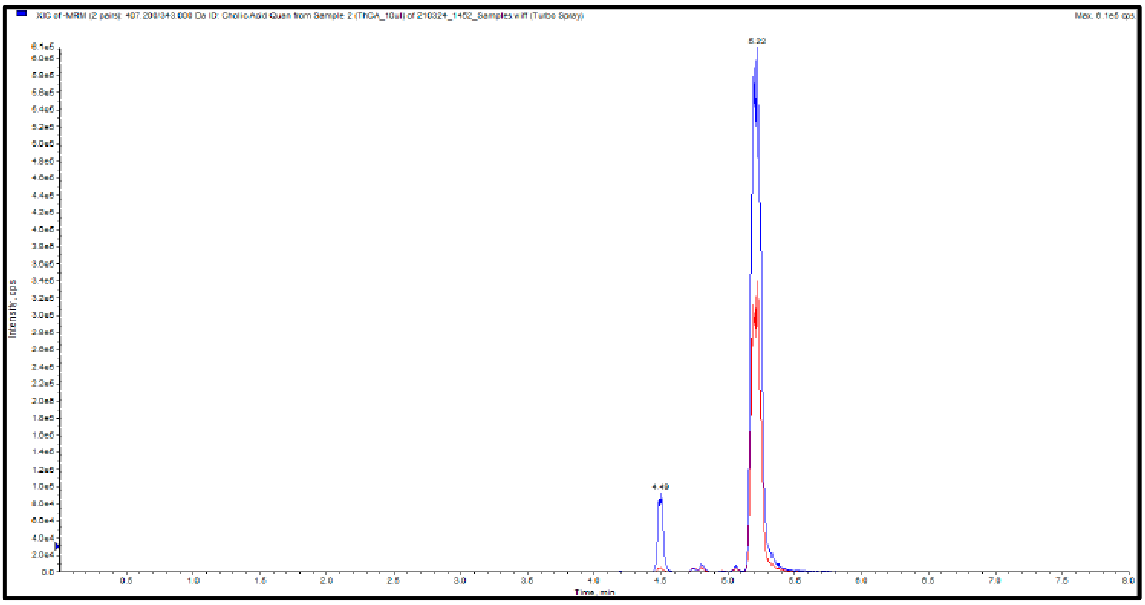

Figure S20: Retention time chromatogram of cholic acid isolated from *T. haliotis* with a retention time of 5.22mins.

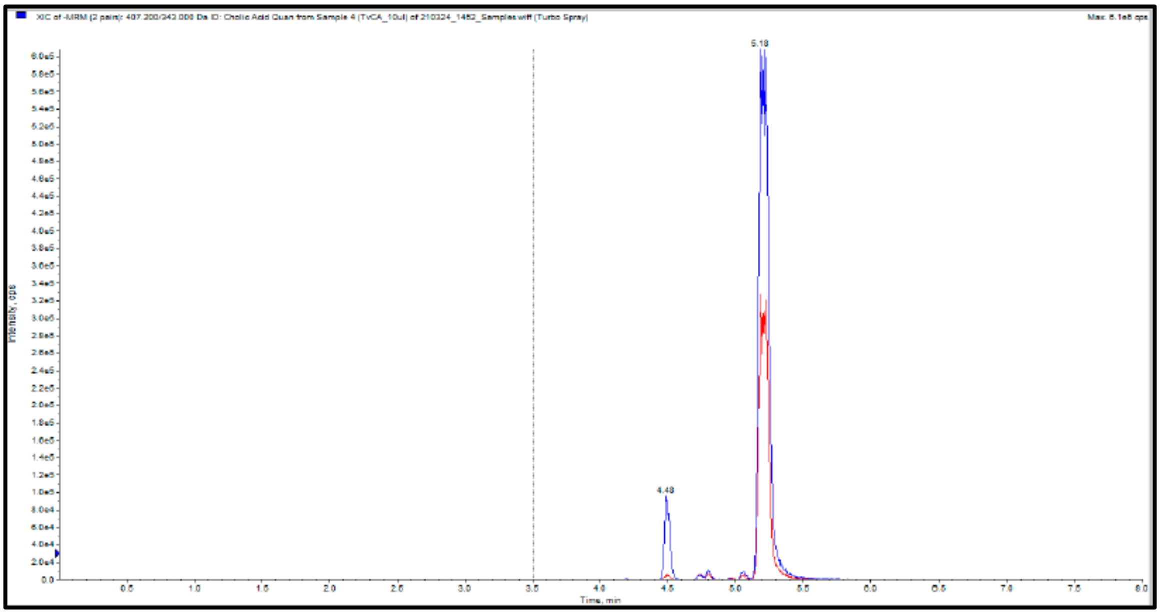

Figure S21: Retention time chromatogram of cholic acid isolated from *T. viridans* with a retention time of 5.18mins.

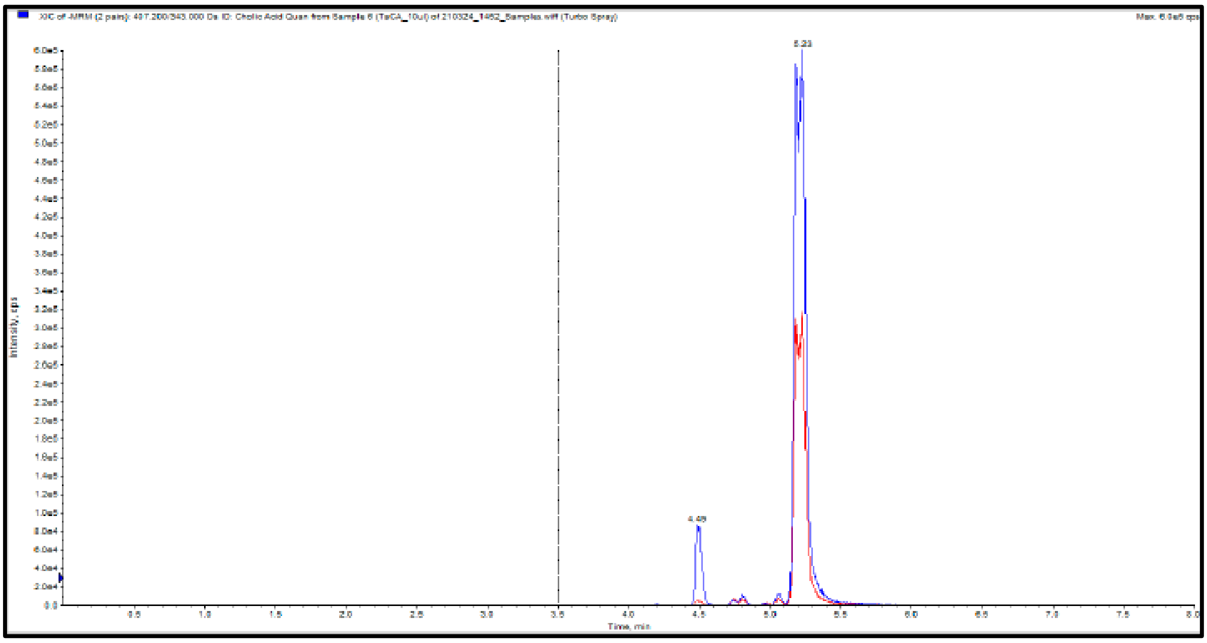

Figure S22: Retention time chromatogram of cholic acid isolated from *T. actiniarum* with a retention time of 5.22mins.

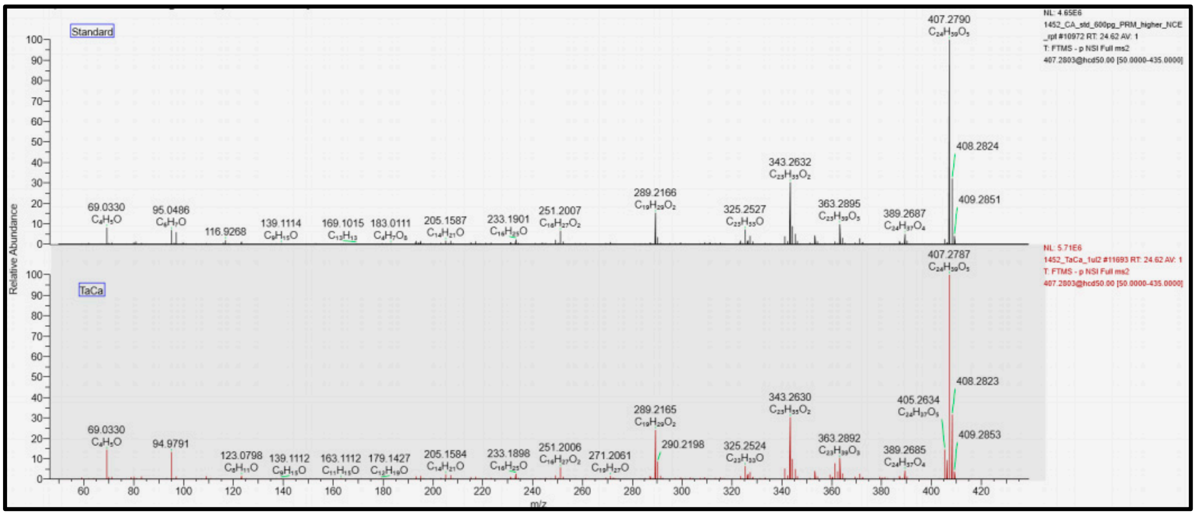

Figure S23: Comparison of full scan (*m*/*z* 50-435) negative-ion mass spectrum of cholic acid (standard) and cholic acid isolated from *T. actiniarum* (using preparative TLC).

```
BAF94155.1         --EQTAFV-SDMQSKLLARAARITTPETNPRDLGSNGWGLGKDKTENGRGMVLGNPHFPH  288 Aac
TACT_08755         -INNTSFI-AGLRQNIRHQADSFTPPNQRHDNLGSNGWGLGKDNTENGRGLVLANPHFPY  283
THAL_08470         -TAHNPLI-ANLQQNVQQRASLFSMPDQEHGDLGSNGWSLGKDKTENGRGLVLANPHFPY  281
TVIR_20855         -PSHDKLL-TGLRQNIRRQAGNFTIPDQEHGDLGSNGWGLGKDKTENGQGILLANPHFPY  282
BAV56778.1         -AFE-PGR-----------TRAPSLQVGGELGVGSNMYGFGTAATGEGSGVLFGNPHWYW  259 MacQ*
AAG05773.1         -SGEQAFQ-----------VAEQ-RRQRFRLERGSNAIAVGSERSADGKGMLLANPHFPW  242 pvdQ
AAY37014.1         -QQAQALQ-----------LAAA-RNQRFALERGSNAVAIGRELSANGRGMLLANPHFPW  249 HacA
WP_010889514.1     --APRPDL-----------AAFNRQYRFNDLPIGSNGWAFGSEATTNGRGLLLGNPHFPW  251 QqaR
AAO41113.1         -PPQAVDT-----------VALDRELQLRDMPIGSNGWAFGADATANRRGVLLGNPHFPW  258 AiiD
WP_011002462.1     -PPHAVDV-----------AALDRELRLRDLPIGSNGWAFGSAATENRRGVLLGNPHFPW  259 RsAac
WP_043523659.1     ---EAPDA-----------AAIAAALDGTSAGIGSNAYGLGAQATVNGSGMVLANPHFPW  251 AuAAC*
BAF51977.1         --VADPAA-----------AARAYFAARHDTGMGSNAVAFSGAATANRRGLLLGNPHYPW  270 SmPVA
AAU09670.1         --PKEAAA-----------AAQ-RLLSTQNADMGSNAVAFRGSTTANGRGLLLGNPHYPW  267 SlPA*
AAT68473.1         --PEEAAS-----------AAK-RLLSAQNADMGSNAVAFDGSTTVNGRGLLLGNPHYPW  265 AhlM*
BAD07025.1         --PEEAAT-----------AAE-RLLSTQNADMGSNAVAFDGSTTVNGRGLLLGNPHYPW  265 SsCLA
TVIR_22915         ---------NGTAALS-GKTQA--QTYQDQALPGSNNWAVSGKLSENGSAILANDMHLNL  285
THAL_06600         ---------DGAAAQT-DKVSANPFSYRDQALPGSNNWAVSGKLSENGSAILANDMHLNL  287
TACT_06920         ---------NSTATHS-GQAQANNSLYRDQALPGSNNWAVSGKLSENGSAILANDMHLNL  287
AAG04421.1         ---------PGLAGVEGAARQVAA-LSML-GVAASNNWAIAPQRSRSGKSLMANDTHLPL  290 QuiP
AAY39885.1         -----------LNQLAQLSHKALE-DAGLPQFEGSNAWVISGSRTQSGKPILAGDPHIRF  273 HacB
BAB75623.1         VIKEVGEIAPVLESIKKPMVALEKLIGHTGVGIGSNNWVISGERTATGKPILANDPHLAV  314 AiiC
AAL53453.1         ---------ISLEELRPAIEGLMKLFPHDATGGGSNNWAVDARHSKTGMPIVAGDPHQAY  252 AibP
TACT_32120         ---------DIILGLLAQNEQVQPQFNVGGEGLGSNAWVVSGEYTQSGLPLLASDPHLGV  281
ASS36259.1         ---------VALENVMRRNSQLQKQFNFGGQYVGSNAWVISGKHSKDNMPILANDPHLGL  281 PfmA*
TVIR_31235         ---------DILTDLLALNHKFEDGLKIGGRFVGSNAWVVSGKLTESGRPILANDPHLGL  284
```

```
BAF94155.1         TGNLRFWQSHITIPG-----QLDMMGGSLVGMPGPINIGFNKDLAWTHTFSTAEHFVMYN  343 Aac
TACT_08755         TGHLRFWQSHLTIPG-----VLDVMGGSLQGLPGVINIGFNQHIAWTHTVSKSRRFVLYQ  338
THAL_08470         TGHLRFWQSHITIPG-----VLDVMGGSLQGLPGIVNIGFNQHLAWTHTVSKSRRFALYQ  336
TVIR_20855         TGHLRFWQSHVTIPG-----VLDVMGGSLQGLPGIVNIGFNQNIAWTHTVSNSRRFVLYQ  337
BAV56778.1         KGPDRFYQAQLTIDG-----EANVSGVSFLGLP-VIQIGFNDSVAWSHTVSTARRFGFFQ  313 MacQ*
AAG05773.1         NGAMRFYQMHLTIPG-----RLDVMGASLPGLP-VVNIGFSRHLAWTHTVDTSSHFTLYR  296 pvdQ
AAY37014.1         GGGMRFYQMHLTIPG-----KLDVMGAALPGLP-LINIGFNQHLAWSHTVDTSKHFTLHR  303 HacA
WP_010889514.1     ETSNRFYQLHLTLPG-----QFDVMGASLGGMP-VVNIGFNQDVAWTHTVSTDKRFTLAA  305 QqaR
AAO41113.1         TTTNRFYQVHLTVPG-----KLDVMGASIAAFP-VVSIGFNKDVAWTHTVSTGRRFTLFE  312 AiiD
WP_011002462.1     TTTNRFYEVHLTVPG-----KLDVMGASIAAFP-VVSIGFNRDVAWTHTVSTGRRFTLFE  313 RsAac
WP_043523659.1     QGAERFYRMHLKVPG-----RYDVEGAALIGDP-IIEIGHNRTVAWSHTVSTARRFVWHR  305 AuAAC*
BAF51977.1         HGNRRFWQSQQTIPG-----ELNVSGASLLGSP-TVQIGHNASMAWSHTVATGVPMNLYE  324 SmPVA
AAU09670.1         DGGRRFWQSQQTIPG-----ELNVAGGSLLGST-TVSIGHNADVAWSHTVATGVTLNLHQ  321 SlPA*
AAT68473.1         QGGRRFWQSQQTIPG-----ELNVSGASLLGAT-TMSIGHNPDVAWSHTVATGVTLNLHQ  319 AhlM*
BAD07025.1         QGGRRFWQAQQTIPG-----ELNVSGASLLGAT-TISIGHNADVAWSHTVATGVTLNLHQ  319 SsCLA
TVIR_22915         RVPNTWYRASLEYKS-HEHEAVKVTGVSLPGTPS-IIAGSNGDIAWGFTNSYGDWSDVIL  343
THAL_06600         RVPNTWYRTSLEYRN-HENQAVKVTGVSLPGAPS-IIAGSNGNIAWGFTNSYGDWSDVIL  345
TACT_06920         RVPNTWYRASLEYRN-HENQAVKVTGVSLPGAPS-IIAGSNGDIAWGFTNSYGDWSDVIL  345
AAG04421.1         SMPSVWNYVQIRSP------KYQAAGVSIAGLPG-VVAGFNGKLAWGMTMVLGDNQDLYL  343 QuiP
AAY39885.1         SVPSVWYEAQLSAP------GFELYG-HFPALNPFALLGHNMDFGWSLTMFQNDDVDLIA  326 HacB
BAB75623.1         QMPSIWYEVALHCIKQTDDCPYNVSGFSFPGMLG-VIVGHSDRIAWGVTNTQGDVMDLYI  373 AiiC
AAL53453.1         EIPGMYAQIHLTCD------SFDVLGLSVPGVPGFPHFAHNEEMAWCVTHAFADIHDLYV  306 AibP
TACT_32120         EIPAVWYLAEIQGD------ELHVTGATYPGAPI-VLMGQNESIAWGTTNMLADAQDLYV  334
ASS36259.1         QIPSYWYALSQQGE------HLSAKGMGLVGTPL-IIFGQNDHISWGGTNMMADVQDLYF  334 PfmA*
TVIR_31235         QIPSLWYAVSQQGD------TVSAAGMSLVGLPL-VIFGKNEHIAWGGTNMMADVQDLYL  337
```

Figure S24: Amino acid sequence alignment of putative penicillin acylases from *Thalassomonas* sp. with AHL acylase homologues. Only regions with conserved residues involved in autoproteolytic cleavage and catalysis are depicted here and residues are highlighted in yellow. The amino acid sequence of the acylases from *Thalassomonas* sp. are depicted in bold. Amino acid sequences in pink depict group A AHL acylases while those in blue depict group B AHL acylases. Acylases with an asterisk are reported to have both AHL acylase and penicillin acylase activity.
